# Integrative analysis of 115 transcriptomic studies decodes the molecular landscape of neurodevelopmental disorders

**DOI:** 10.1038/s42003-025-08330-2

**Published:** 2025-06-12

**Authors:** Jarno Koetsier, Lars M. T. Eijssen, Leon J. Schurgers, Leopold M. G. Curfs, Chris P. Reutelingsperger, Nasim Bahram Sangani

**Affiliations:** 1 Department of Biochemistry, Cardiovascular Research Institute Maastricht (CARIM), Maastricht University, Maastricht, The Netherlands; 2 GKC, Maastricht University Medical Centre, Maastricht, The Netherlands; 3 Department of Psychiatry and Neuropsychology, School for Mental Health and Neuroscience (MHeNs), Maastricht University, Maastricht, The Netherlands; 4 Department of Translational Genomics, Maastricht University, Maastricht, The Netherlands

**Keywords:** Data integration, Developmental disorders

## Abstract

Due to the low disease prevalence, transcriptomic studies of neurodevelopmental disorders (NDDs) often face limited statistical power, constraining the depth of insights they can provide. To tackle this limitation, we integrated 151 human RNA sequencing datasets from 115 independent studies, and characterized the common and distinct molecular pathways of NDDs and their neurological phenotypes. In addition to revealing an aberrant expression profile of imprinted genes, our analysis identified transcriptomic changes in inflammatory, translational, mitochondrial, and synaptic processes across the different NDDs. We further highlight disorder-associated alterations, including upregulation of *ITGB4* across Rett syndrome datasets. Moreover, gene expression changes in *LHX1/5*-mediated cerebellar Purkinje cell layer formation were found to be specific to seizure-associated NDDs. We combined the datasets into a publicly accessible NDD transcriptomic atlas: https://SyNUM.shinyapps.io/NDD-transcriptomic-atlas/. Together, our findings provide fundamental insights into the molecular pathophysiology of NDDs and highlight genes and pathways with aberrant transcriptomic profiles. This knowledge can guide future therapeutic development and precision medicine approaches.

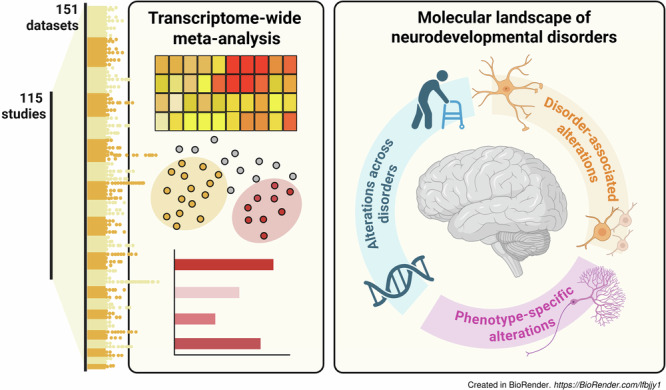

## Abstract (web summary)

Integrative analysis of 115 transcriptomic studies reveals shared and unique molecular pathways underlying neurodevelopmental disorders and their associated neurological phenotypes.

## Introduction

Neurodevelopmental disorders (NDDs) are characterized by a disrupted brain development, leading to a wide range of psychiatric and neurological conditions that affect more than 4.7% of children worldwide^1,2^. These conditions typically emerge in childhood^2^ and include rare genetic disorders, such as Rett syndrome (RTT), Fragile X syndrome (FXS), and Duchenne muscular dystrophy (DMD), as well as multifactorial conditions, such as attention deficit hyperactivity disorder (ADHD) and autism spectrum disorder (ASD)^3^. Despite the wide range of clinical manifestations, many neurological phenotypes are shared across distinct NDDs, indicating the involvement of common molecular pathways. Examples of such shared phenotypes include, among others, seizures, intellectual disability, microcephaly, and hypotonia.

High-throughput techniques, such as RNA sequencing, have significantly advanced our understanding of the molecular pathways involved in NDDs. By identifying genes and pathways with altered expression levels, transcriptomic profiling provides insights into the pathological mechanisms and enables the discovery of potential therapeutic targets. The importance of expression profiling for therapeutic target discovery is exemplified by the observation of brain-derived neurotrophic factor (BDNF) downregulation in RTT in the early 2000’s^4^. Since BDNF cannot cross the blood-brain-barrier, this discovery led to the prioritization of insulin-like growth factor 1 (IGF1), a factor with similar biological properties as BDNF, as a promising therapeutic candidate^5^. More recently, IGF1 treatment (Trofinetide) has been FDA-approved for the treatment of RTT^6^, highlighting the potential of expression profiling for the identification of therapeutic targets.

Nevertheless, current transcriptomic studies of NDDs are often constrained by small sample sizes or high biological variability, particularly for rare genetic disorders and conditions with a complex disease etiology, respectively. Because of these limitations, these studies do not achieve the statistical power needed to fully characterize the disease transcriptome and to uncover novel molecular pathways. Integrating multiple datasets can increase statistical power, allowing for deeper insights into the molecular pathophysiology of NDDs. In our study, we therefore integrated 151 human RNA sequencing datasets from 115 independent studies to characterize common and distinct molecular pathways of NDDs and their neurological phenotypes.

## Results

### The NDD transcriptomic profile consists of 151 datasets from 115 independent studies

**Fig. 1:**
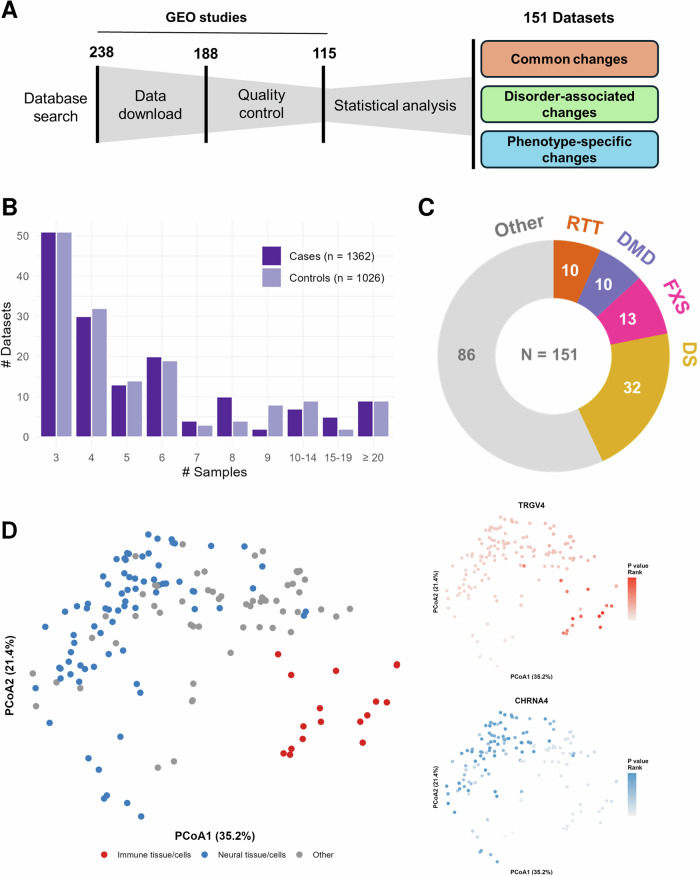
Overview of the study. **A** Workflow of data collection and analysis. Of the 238 datasets identified by the search query, 188 datasets included at least six samples and had NCBI-generated RNA sequencing count data available. Of these datasets, those without case-control design, with less than three cases and/or controls, and without NDD cases were excluded, resulting in 115 transcriptomic datasets that were included in the analysis. Datasets were stratified based on mutation type and/or cell type/tissue before performing differential expression analysis, provided that there were at least three cases and controls within each stratum. This resulted in a total of 151 distinct datasets that were used to identify common, disorder-associated, and phenotype-specific changes. **B** Distribution of the number of cases and controls among the 151 datasets. The y-axis shows the number of datasets with the specified number of cases and controls on the x-axis. Most datasets include only three or four cases and controls, while only a few datasets include more than ten. **C** Donut chart of the number of datasets for Rett syndrome, Duchenne muscular dystrophy, Fragile X syndrome, Down syndrome, and others. **D** Principal coordinate analysis (PCoA) of the 151 datasets. The distance between the datasets was calculated using the Spearman correlation of the gene’s *P* values. The gene’s *P* values were calculated for each dataset through the differential expression analysis of NDD cases versus controls. The first component is associated with the cell type/tissue. Particularly, the T Cell Receptor Gamma Variable 4 (TRGV4) reaches higher levels of significance (i.e., *P* value rank) in immune cells, while the cholinergic receptor nicotinic alpha 4 Subunit (CHRNA4) has higher *P* value ranks in neural cells.

The Gene Expression Omnibus (GEO) was queried for RNA sequencing data of NDDs (Supplementary Text S1), identifying 188 studies with NCBI-generated raw counts available for at least six samples. Datasets without case-control design, with less than three cases and/or controls, and without NDD cases were excluded. The 115 studies that remained after filtering were included in our analysis (Supplementary Data S1). Where possible, individual studies were stratified based on mutation type and/or cell type/tissue before performing differential expression analysis (i.e., case versus control), yielding a total of 151 distinct datasets/statistical comparisons. The differential expression estimates of the datasets were used to identify general transcriptomic changes that occur across the different NDDs and to find alterations that are associated with a specific disorder or neurological phenotype (Fig. 1A). The summary statistics of the datasets can be downloaded and interactively explored on our website (https://SyNUM.shinyapps.io/NDD-transcriptomic-atlas/).

Most of the 151 datasets encompassed only three or four cases and/or controls (Fig. 1B). The most common NDDs in our meta-analysis include RTT, FXS, DMD, and down syndrome (DS), which together account for 43% of all datasets (Fig. 1C). The causative genes of these four NDDs (i.e., *MECP2*, *FMR1*, *DMD*, and chromosomal 21 genes, respectively) exhibited the anticipated transcriptomic alterations (Supplementary Fig. S1). Moreover, datasets clustered mostly by cell type/tissue rather than by disease, highlighting the importance of tissue choice in transcriptomic experiments (Fig. 1D, Supplementary Fig. S2). This is exemplified by cholinergic receptor *CHRNA4* and T-cell receptor *TRGV4,* which only reach high levels of significance in neural and immune cell types/tissues, respectively.

### NDDs are characterized by inflammatory, translational, mitochondrial, and synaptic alterations

**Fig. 2:**
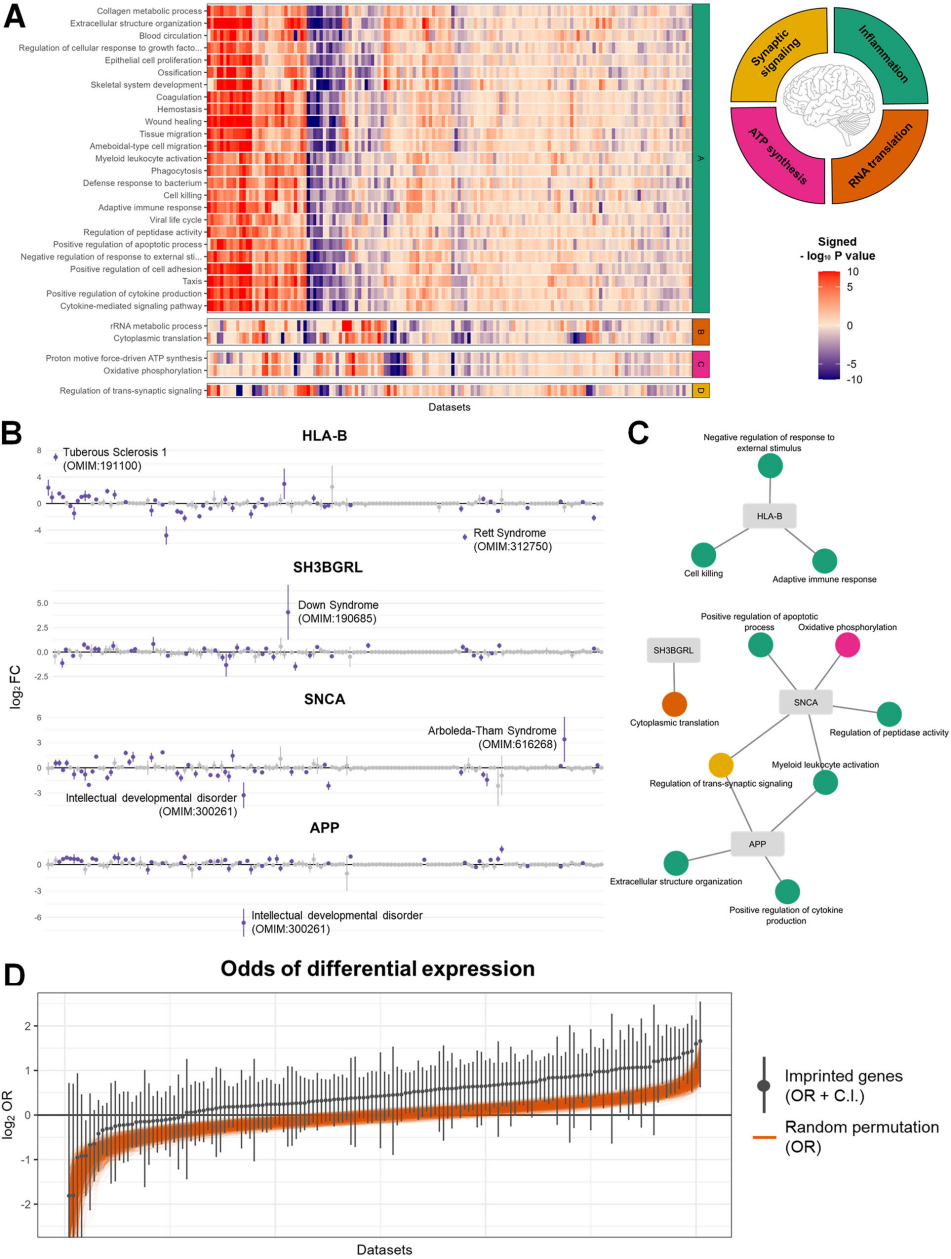
Common alterations of the neurodevelopmental disorders (NDDs). **A** Heatmap of the enrichment of the top 30 most frequently enriched Gene Ontology – Biological Process (GO-BP) terms across the 151 NDD datasets. These GO-BP terms fall mostly within four categories: inflammation, RNA translation, ATP synthesis, and synaptic signaling. **B** To exemplify gene-level transcriptomic alterations across 151 NDD datasets, this panel shows the log_2_FC profile of the most frequently differentially expressed gene for the GO-BP terms related to inflammation, RNA translation, ATP synthesis, and synaptic signaling (*i.e., HLA-B*, *SH3BGRL*, *APP*, and *SNCA*, respectively). Colored data points indicate statistical significance (*P* value < 0.05). The log_2_FC profiles of *HLA-B*, *SH3BGRL*, and *SCNA* are not associated with any specific disorder, phenotype, sample type, or model system (independent two-group Mann-Whitney *U* Test, FDR-adj. *P* value > 0.05, Supplementary Fig. S6). However, because of its location on chromosome 21, *APP* exhibits higher log_2_FCs in Down syndrome than in any other disorder (independent two-group Mann-Whitney *U* Test, FDR-adj. *P* value = 3.9e-7). Nevertheless, after excluding Down syndrome datasets, *APP* is still among the top 10% most frequently differentially expressed genes in the *“Regulation of trans-synaptic signaling”* GO term. **C** Network linking the genes (see panel B) to their associated GO-BP terms (**A**). **D** The log_2_ Odds Ratio (log_2_ OR) and the 95% confidence interval (C.I) of differential expression (*P* value < 0.05) of imprinted versus non-imprinted genes as calculated by the Fisher’s exact test. Imprinted genes have higher odds of being differentially expressed than non-imprinted genes (log_2_ OR > 0) in 123 datasets. The orange lines show the ORs of 1000 random gene sets with the same size and expression profile as the imprinted genes, demonstrating that the higher odds of differential expression of imprinted genes occurs more than expected by chance (permutation *P* value < 0.001).

Our first aim was to identify transcriptomic alterations that are common across NDDs. In particular, gene set enrichment analysis (GSEA) was performed on the 151 datasets to find Gene Ontology–Biological Process (GO-BP) terms with a differential expression profile across the NDDs. The 30 GO-BP terms that reached statistical significance (i.e., false discovery rate-adjusted (FDR-adj) *P* value < 0.05) across most NDD datasets encompassed processes related to inflammation, RNA translation, mitochondrial ATP synthesis, and synaptic signaling (Fig. 2A, Supplementary Fig. S3, and Supplementary Data S2). The GSEA of molecular function and cellular component show similar results and are provided in Supplementary Fig. S4.

Notably, enrichment of the GO-BP terms (i.e., -log_10_ *P* value of the GSEA) was not associated with any specific NDD or neurological phenotype (independent two-group Mann-Whitney *U* Test, FDR-adj. *P* value > 0.05, Supplementary Fig. S5), suggesting that these transcriptomic changes are not driven by a specific disorder or phenotype but instead are shared across the NDD spectrum. There were neither any significant associations between the GO-BP enrichment and the sample type (i.e., immune, neural, and other cell types/tissues) or model system (i.e., in vitro and non-in vitro models) (Supplementary Fig. S5**)**. Among the genes in the inflammation-, RNA translation-, ATP synthesis-, and synaptic signaling-related GO-BP terms, *HLA-B*, *SH3BGRL*, *APP*, and *SNCA*, respectively, were differentially expressed in the largest number of datasets, exemplifying gene-level transcriptomic alterations across NDDs (Fig. 2B-C and Supplementary Fig. S6). Moreover, changes in ATP synthesis were linked to a strongly dysregulated expression of mitochondrial-encoded genes (Supplementary Fig. S7).

### Imprinted genes exhibit a dysregulated transcriptomic profile in NDDs

Since imprinted genes are known to have essential roles during brain development^7^, their dysregulation might be involved in NDD pathology. In line with this hypothesis, we observed large transcriptomic alterations in imprinted genes, such as *PEG10* and *MEST*, which were differentially expressed in 27% and 24% of the NDD datasets, respectively (Supplementary Fig. S8). More generally, in 123 datasets, imprinted genes were found to have higher odds of differential expression than non-imprinted genes, which is more than expected by chance (permutation *P* value < 0.001, Fig. 2D). This preferential differential expression of imprinted genes was observed for datasets of in vitro (permutation *P* value < 0.001) and non-in vitro (permutation *P* value = 0.003) systems (Supplementary Fig. S9). Furthermore, imprinted genes exhibited higher odds of both upregulation and downregulation (Supplementary Fig. S10).

### RTT, DMD, DS, and FXS have distinct transcriptomic alterations

**Fig. 3:**
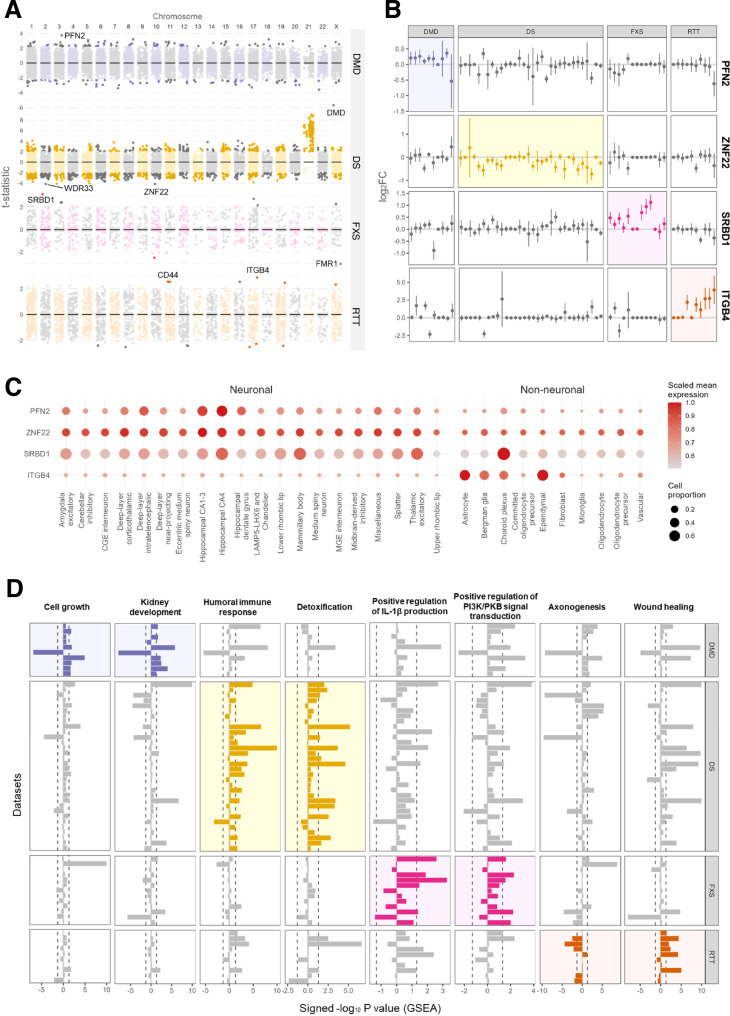
Disorder-associated transcriptomic alterations. **A** The genes’ t-statistics of the meta-analysis for the DMD, DS, FXS, and RTT datasets. In the meta-analysis, the log_2_FC estimates and their standard errors were used to calculate the joint t-statistics. **B** The log_2_FCs of *PFEN2*, *ZNF22*, *SRBD1*, and *ITGB4* across the DMD, DS, FXS, and RTT datasets. *PFEN2*, *ZNF22*, *SRBD1*, and *ITGB4* are the genes that are most strongly associated with DMD, DS, FXS, and RTT, respectively. **C** Expression of the four gene marker per supercluster in the brain (Human Brain Cell Atlas v1.0). The color scale shows the mean expression of the expressing cell fraction. This value is scaled per row as the faction of the maximum value. **D** Bar chart of the signed -log_10_ *P* value for the disorder-associated GO-BP terms. The GO-BP terms shown for each disease are the two terms that are significantly enriched (*P* value < 0.05) with consistent direction of effect in the largest number of DMD, DS, FXS, and RTT datasets (from top to bottom).

To identify disorder-associated transcriptomic changes, separate transcriptome-wide meta-analyses on the log_2_ fold change (log_2_FC) estimates (random effect, inverse variance method, Hartung-Knapp adjustment) were performed for DMD, DS, FXS, and RTT. After excluding the causative genes, the most significantly differentially expressed genes in the meta-analysis for these four disorders were *PFN2*, *ZNF22*, *SRBD1*, and *ITGB4*, respectively (Fig. 3A-B, Supplementary Data S3). Of these genes, *PFN2* is mostly expressed in hippocampal neurons (Human Brain Cell Atlas^8^ version 1.0, Fig. 3C). In contrast, *ITGB4* is not expressed in neuronal cells, but is specific to glia such as astrocytes and ependymal cells. Additionally, by performing GSEA, several processes with differential expression profiles in DMD, DS, FXS, and RTT were found (Fig. 3D). For instance, *axonogenesis* was downregulated in RTT and *humoral immune response* was upregulated in DS.

### *LHX1/5*-related Purkinje cell layer formation is altered in seizure-associated NDDs

**Fig. 4:**
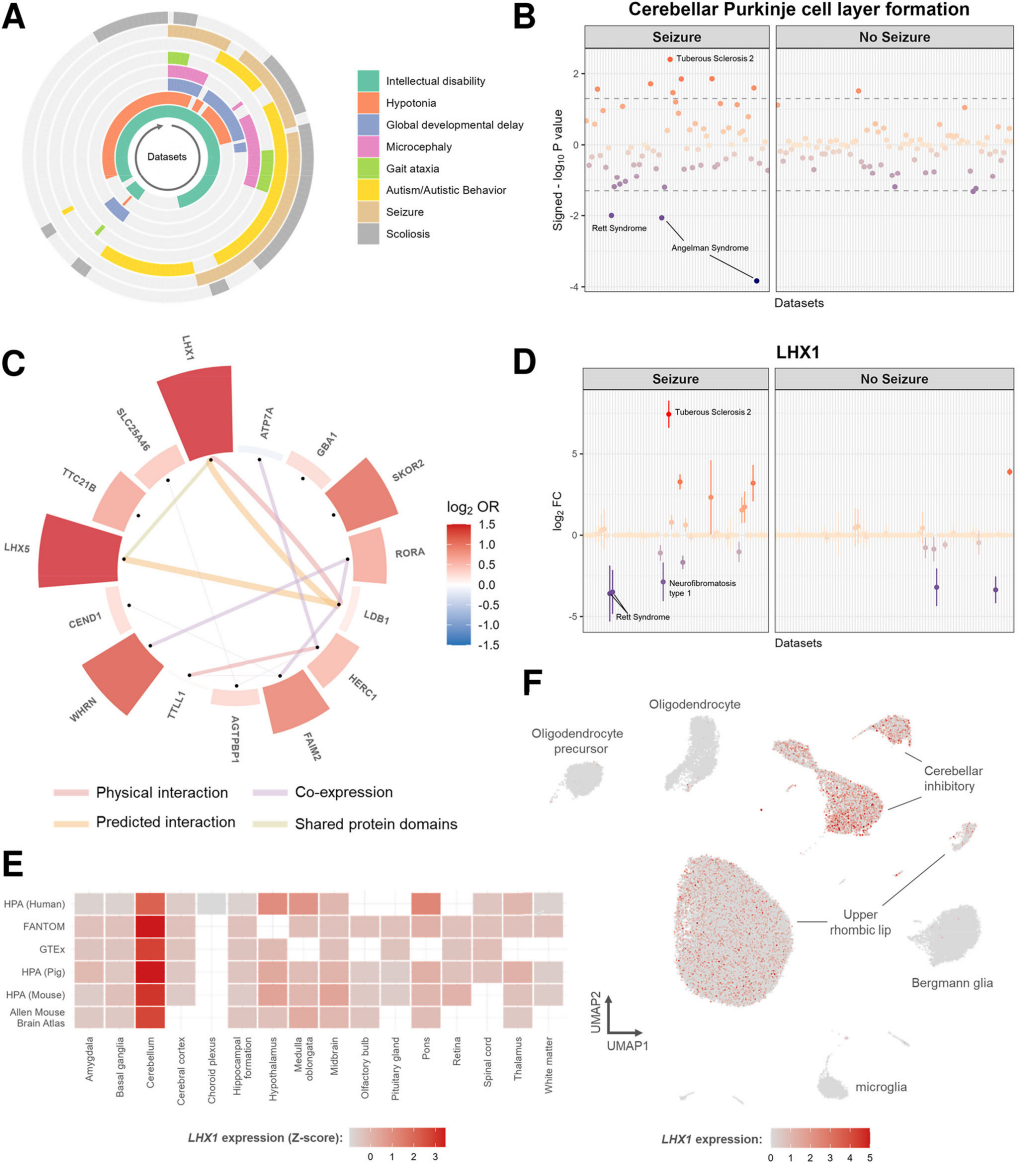
Phenotype-specific transcriptomic alterations. **A** Circular heatmap of the distribution of the eight neurological phenotypes across the 151 NDD datasets. **B** The signed -log_10_ *P* value of the *Cerebellar Purkinje cell layer formation* GO-BP term across the different datasets. A negative and positive signed -log_10_ *P* value were calculated through Gene Set Enrichment Analysis (GSEA) and indicates down- and upregulated expression, respectively. **C** log_2_ odds ratio (OR) of differential expression in seizure- versus non-seizure-associated neurodevelopmental disorders for all genes associated with cerebellar Purkinje cell layer formation. *LHX1* has the highest OR. The lines indicate different types of protein-protein and gene-gene relationships from GeneMANIA (version 3.5.3). **D** log_2_FC profile and their 95% confidence interval of the *LHX1* gene in seizure- and non-seizure-associated neurodevelopmental disorders. **E** *LHX1* expression levels across different brain regions in six different datasets available from the Human Protein Atlas: HPA (Human), FANTOM, GTEx, HPA (Pig), HPA (Mouse), and Allen Mouse Brain Atlas. The Z-score was calculated for each dataset separately. **F** UMAP plot of the cerebellar vermis scRNA-seq data from the Human Brain Atlas v1.0. The *LHX1* expression in the different superclusters is shown. Specifically, 51 and 28% of the cerebellar inhibitory and upper rhombic lip cells, respectively, express *LHX1*.

The final aim was to identify transcriptomic alterations in genes and biological processes that are associated with specific NDD phenotypes. Using the Human Phenotype Ontology^9^, the NDDs were linked to eight distinct phenotypes: intellectual disability, hypotonia, global developmental delay, microcephaly, gait ataxia, autism/autistic behavior, seizure, and scoliosis (Fig. 4A). Interestingly, the differential expression of *cerebellar Purkinje cell layer formation* was strongly correlated with seizure-associated NDDs (FDR-adj. *P* value = 0.01, Fig. 4B). Within this GO-BP term, the LIM homeobox genes *LHX1* and *LHX5* had a higher likelihood of differential expression (*P* value = 0.01 and 0.03, respectively) in NDDs associated with seizures compared to those without seizures (Fig. 4C-D, Supplementary Fig. S11).

Publicly available single-cell RNA sequencing (scRNA-seq) data from first trimester developing human brain^10^ showed that during early neurodevelopment, both *LHX1* and *LHX5* are mostly expressed in the hindbrain and midbrain regions (Supplementary Fig. S12). In the adult brain (Human Protein Atlas^11^ v24.0), *LHX1* expression is highly specific to the cerebellum (Fig. 4E). This is in contrast to *LHX5* which is also abundantly expressed in the hypothalamus and medulla oblongata (Supplementary Fig. S13). Moreover, scRNA-seq data of the cerebellar vermis (Human Brain Atlas^8^ v1.0) revealed that *LHX1* is almost exclusively transcribed in cerebellar neurons, particularly in those of the cerebellar inhibitory supercluster (Fig. 4F).

Besides *cerebellar Purkinje cell layer formation*, no other GO-BP term’s differential expression profile was significantly associated with any of the eight neurological phenotypes (i.e., FDR-adj. Mann-Whitney U Test *P* value > 0.05). Nevertheless, several genes, including *ETS2*, *MRPS6*, *PIGP*, and *UBE2G2*, were found to be more likely upregulated (i.e., *P* value < 0.05 and log_2_FC > 0) in hypotonia-associated NDDs than in NDDs without hypotonia (FDR-adj. Fisher’s exact test *P* value < 0.05, Supplementary Fig. S14 and Supplementary Data S4).

## Discussion

In this study, we conducted a transcriptome-wide meta-analysis of 151 human RNA sequencing datasets from 115 independent studies. Through this analysis, we revealed the aberrant transcriptomic profile of imprinted genes across NDDs. Additionally, we identified NDD-wide expression changes in inflammatory, translational, mitochondrial, and synaptic functions. Disorder-specific alterations were also uncovered, with *PFN2*, *ZNF22*, *SRBD1*, and *ITGB4* showing differential expression in DMD, DS, FXS, and RTT, respectively. Finally, we showed that transcriptomic changes related to cerebellar Purkinje cell layer formation—in particular the differential expression of *LHX1* and *LHX5*—are specific to seizure-associated NDDs.

Across most NDD datasets, imprinted genes were found to be more likely to be differentially expressed than non-imprinted genes (Fig. 2D). Genetic defects in some imprinted genes are well-known causes of NDDs. For example, Angelman and Prader-Wili syndromes are linked to the imprinted 15q11-q13 region, while Temple and Kagami-Ogata syndromes are associated with mutations or uniparental disomy of the imprinted 14q32 region^12^. Moreover, genes from the 14q32 region, including the *chromosome 14 miRNA cluster*, *MEG3*, and *MEG8*, have also been linked to RTT pathology. Particularly, previous studies on RTT found differential expression of 14q32 genes or their murine homologs in brain organoid-derived extracellular vesicles^13^ and mouse brains^14,15^. Besides RTT, imprinted genes are involved in the pathology of other NDDs, such as Williams syndrome^16^, fetal alcohol syndrome^17^, and ASD^18^. Until now, the link between NDDs and imprinted genes has been based on these specific examples. Our findings, however, provide the first systematic evidence for the dysregulation of imprinted genes across a wide range of NDDs. This widespread dysregulation can be explained by the essential roles of imprinted genes in the regulation of neural proliferation, differentiation, and migration^7^. Particularly, any alteration in these tightly regulated processes may impair normal brain development, giving rise to NDD phenotypes.

We further identified synaptic signaling as one of the processes with transcriptomic alterations across the various NDD datasets (Fig. 2A). Likewise, mutations in synaptic genes are known to give rise to a wide range of NDD phenotypes, including hypotonia, intellectual disability, ataxia, and developmental delay^19^. Among synaptic signaling-associated genes, we found large expression changes in *APP* and *SNCA* (Fig. 2B-C), which have been linked to familial Alzheimer’s^20^ and Parkinson’s disease^21^, respectively. In line with previous observations^22^, these findings point towards similarities in synaptic dysfunction between neurodevelopmental and neurodegenerative disorders.

Transcriptomic alterations in oxidative phosphorylation (Fig. 2A) and mitochondrial-encoded genes (Supplementary Fig. S7) may indicate the presence of mitochondrial dysfunction across the various NDDs. Indeed, many NDDs, including RTT, FXS, Angelman syndrome, and tuberous sclerosis complex, are characterized by dysfunctional mitochondria^23^. The shared involvement of mitochondria relates to the essential role of mitochondrial dynamics during neurogenesis and synaptogenesis^24^. Moreover, mitochondrial function, in particular the shift to oxidative phosphorylation, is required for the induction of neuronal differentiation^25^.

Through the excessive production of reactive oxygen species or the release of danger-associated molecular patterns, mitochondrial dysfunction can trigger (neuro)inflammation^26^, which, just as oxidative phosphorylation, exhibited transcriptomic alterations across the NDD datasets (Fig. 2A). Like mitochondrial dysfunction, aberrant inflammatory responses during neurodevelopment are considered key processes in NDD pathology^27^. Interestingly, inflammation can, in turn, compromise mitochondrial function, including oxidative phosphorylation^26^.

It is interesting to note that processes exhibiting transcriptomic changes across NDDs, including mitochondrial and synaptic function, RNA translation, and inflammatory processes, closely resemble hallmarks of ageing (i.e., mitochondrial dysfunction, altered intercellular communication, loss of proteostasis, and chronic inflammation, respectively). This observation may suggest that, at the molecular level, NDDs represent a state of *accelerated* or *early-onset* ageing. This hypothesis is supported by the fact that many NDDs have an associated neurodegenerative phenotype: *the neurodevelopmental-degenerative continuum*^28^. For instance, DS and FXS are strongly associated with the development of Alzheimer’s disease and movement disorders, respectively. Particularly, almost all individuals with DS will have been diagnosed with Alzheimer’s disease by the age of 70 years, with a median age of diagnosis of around 50 years^29^. Moreover, at an age of 40 years or older, nearly 40% of male FXS patients will suffer from movement disorders, such as Parkinson’s disease, tremor, and/or bradykinesia^30^. Together, our findings highlight the need for dedicated analyses assessing the possible overlap in the molecular pathophysiology of neurodevelopmental and neurodegenerative disorders, and its potential for therapeutic interventions.

In our disorder-specific meta-analysis, we identified several transcriptomic markers for DMD, DS, FXS, and RTT (Fig. 3). The identification of these transcriptionally dysregulated genes may provide insights into key pathways downstream of their underlying genetic alterations. Among the identified markers, the upregulation of *PFN2* and *ITGB4* across the DMD and RTT datasets, respectively, may relate to known disease mechanisms, which are elaborated upon in the paragraphs below.

*ITGB4* is highly expressed in astrocytes (Fig. 3C). In these cells, ITGB4 mediates exosome secretion, which, in turn, enhances oligodendrocyte progenitor cell (OPC) proliferation^31,32^. Although OPC proliferation has not been studied in the context of RTT, previous studies did identify differential protein expression of oligodendrocyte markers in the brain of MeCP-null mice^33^. Interestingly, increased expression of the oligodendrocyte marker PLP could not be restored in MeCP2-null mice with MeCP2-expressing oligodendrocytes, possibly due to non-cell autonomous effects^33^. An example of such a non-cell autonomous effect in RTT pathology could be the enhanced ITGB4-mediated exosome secretion by astrocytes.

In DMD, the interaction between actin filaments and the extracellular matrix is lost as a result of dystrophin deficiency^34^. In smooth muscle cells, actin polymerization has been shown to promote dystrophin expression^35^ and strengthen the response to mechanical stress^36^. Hence, upregulation of *PFN2 (*Fig. 3A*)*, an important regulator of actin polymerization^37^, might be a compensatory response aiming at strengthening the weakened interaction between actin filaments and the extracellular matrix in DMD pathology. It should be noted that, although DMD is predominantly characterized by muscular degeneration, dystrophin deficiency also has a significant impact on the electrophysiological function of hippocampal neurons^38^ where *PFN2* is highly expressed (Fig. 3C).

Our phenotype-specific analysis suggests the presence of altered cerebellar Purkinje cell layer formation in seizure-associated NDDs (Fig. 4B). Within this process, we identified a prominent role of *LHX1* and *LHX5*, which showed a dysregulated expression profile specific to seizure-associated NDDs (Fig. 4C, D). Previous studies have shown essential roles of *LHX1* and *LHX5* in the formation of a functional cerebellar Purkinje cell layer^39,40^. For instance, knockout of *Lhx1* and *Lhx5* during embryonic development was shown to inhibit differentiation of Purkinje cell precursors into their mature form, thereby reducing the Purkinje cell pool in the cerebellum^39^. Furthermore, postnatal knockout of these genes limits dendrite development of cerebellar Purkinje cells without reducing the cell number^40^. Although *LHX1* and *LHX5* have not been directly linked to seizures or epilepsy, cerebellar Purkinje cell numbers are decreased in epileptic disorders^41^. Specifically, Purkinje cells are GABAergic neurons that provide inhibitory stimuli to the deep cerebellar nuclei^42^. Through this inhibitory pathway, cerebellar Purkinje cells prevent a seizure-causing hyperexcitability state of the cerebellum. In neurodegenerative disorders, seizures are thought to result from Purkinje cell degeneration or disruptions in Purkinje cell activity^42,43^. Our analysis suggests an alternative mechanism in NDDs, where the dysregulated *LHX1* and *LHX5* expression may contribute to seizure onset by impairing the formation of the Purkinje cell pool. As the negative impact of *Lhx1/5* dysregulation on cerebellar Purkinje cell function extends postnatally^40^, therapies that aim at maintaining physiological levels of LHX1 and LHX5 in the cerebellum may be beneficial for the treatment or management of seizures in NDD. However, it should be noted that *LHX1* and *LHX5* have multifaceted roles during the development of the brain, including regulation of axonal guidance and neural survival^44,45^. Hence, their dysregulation impacts processes beyond Purkinje cell layer formation. Some of these may also contribute to seizures in NDDs—an aspect that warrants further investigation in dedicated future studies.

By integrating and analyzing 151 RNA sequencing datasets from 115 NDD studies, our research achieved statistical power that surpassed that of conventional RNA sequencing experiments, enabling the identification of transcriptomic alterations that have not been described before. Particularly, we highlighted the presence of ageing-related transcriptomic alterations and the differential expression of imprinted genes across distinct NDDs. We further identified multiple disorder- and phenotype-specific changes, such as the upregulation of *ITGB4* in RTT and the differential expression of LIM homeobox genes *LHX1* and *LHX5* in seizure-associated NDDs. The current study is, however, limited to insights at the RNA level. Therefore, future studies should investigate whether the identified alterations are persistent at the protein level and which epigenetic processes are responsible for driving these transcriptomic changes. Furthermore, to translate the insights into cause-consequence relationships and potential therapeutic targets, functional studies, such as knockout experiments, will be needed.

To support other studies to build upon our findings, we combined our data into an atlas that summarizes the current state of knowledge from NDD transcriptomic experiments. The transcriptomic atlas is publicly accessible for exploration and download (https://SyNUM.shinyapps.io/NDD-transcriptomic-atlas/). This resource allows researchers to examine the differential expression profiles of genes of interest across multiple NDDs, serving as a tool to prioritize potential drug targets and streamline the drug discovery process.

## Methods

### Data collection

An overview of the methodological workflow is illustrated in Fig. 1A. The list of NDDs from D’Souza et al.^3^ was used to query the GEO^46^ for RNA sequencing data (NCBI-generated raw count files; Homo sapiens; date of extraction: July 5, 2024). The exact search query is provided in Supplementary Text S1. GEO studies with less than six samples or without NCBI-generated raw count files were excluded from data processing.

### Data processing and statistical analysis

The collected datasets were manually curated and processed. During curation, datasets without case-control design, with less than three cases and/or controls, and without NDD cases were excluded. For the statistical analysis, the *DESeq2*^47^ (version 1.42.0) R/Bioconductor package was applied to compare the RNA expression levels between NDD cases and controls. Samples were stratified by mutation type and/or tissue/cell type before the differential expression analysis, provided that there were at least three cases and controls within each stratum. This means that for some GEO studies more than one statistical comparison has been performed. Furthermore, when information about sex, age/developmental stage, donor, and/or cell type/tissue was available, these factors were included as covariates in the statistical model. The exact experimental design for each dataset is detailed in Supplementary Data S1.

### Disease-phenotype associations

The Human Phenotype Ontology^9^ (version 2.0.4) was used to determine whether the included NDDs were associated with each of the following phenotypes: intellectual disability, hypotonia, global developmental delay, microcephaly, gait ataxia, autism/autistic behavior, seizure, and scoliosis. Each disorder was linked to the relevant phenotypes using its OMIM record in the Human Phenotype Ontology database. When the OMIM record was not available, the ORPHA record was used as an alternative.

### Principal coordinate analysis

Principal coordinate analysis (PCoA) was applied to assess the similarity between the different datasets. The similarity between two datasets *i* and *j* (*S*_*i,j*_) was defined as the Spearman correlation between the datasets’ *P* values (Eq. 1). The distances (*D*_*i,j*_) were calculated from the dataset similarities (Eq. 2) and was double-centered before eigendecomposition.

1 $${S}_{i,j}={Spearman}\left({P}_{i},{P}_{j}\right)$$

2 $${D}_{i,j}=1-{S}_{i,j}$$

### Gene set enrichment analysis

For each statistical comparison, GSEA was performed using the *clusterProfiler*^48^ (version 4.10.1) R/Bioconductor package. For this, GO terms were used as gene sets and the log_2_ fold change (log_2_FC) was used as ranking variable. The *rrvgo*^49^ (version 1.14.2) R/Bioconductor package was applied to cluster similar GO terms using Resnik similarity (threshold = 0.85). The GO term that reached statistical significance (FDR-adjusted *P* value < 0.05) in the largest number of datasets was selected as the representative term for the cluster. For each GO-BP term, the *P* value and enrichment score were used to calculate the signed -log_10_ *P* value according to Eq. 3.

3 $${Signed}{{{{-}}}\log }_{10} \, P{ \, value}={{{-}}}{\log }_{10} \, P \, {value}\cdot {sign}\left({enrichment\; score}\right)$$

### Identification of common NDD transcriptomic alterations

Commonly affected biological processes were identified by finding the GO-BP terms with the largest number of significant NDD datasets (FDR-adjusted *P* value < 0.05). The top 30 GO-BP terms were clustered based on their signed -log_10_ *P* value using hierarchical clustering (Euclidean distance and Ward D2 linkage). Independent two-group Mann-Whitney *U* Test was used to test whether the top 30 GO-BP terms (i.e., signed and unsigned -log_10_ GSEA *P* value) were associated with any of NDDs with at least ten included datasets (i.e., RTT, FXS, DMD, and DS) or common neurological phenotypes (i.e., intellectual disability, hypotonia, global developmental delay, microcephaly, gait ataxia, autism/autistic behavior, seizure, and scoliosis).

Furthermore, it was tested whether imprinted genes play a role in NDD pathology. For this, the Fisher’s exact test was applied to assess whether previously reported or predicted imprinted genes (obtained from *geneimprint.com* on December 5, 2023) have higher odds of differential expression compared to non-imprinted genes. To establish the baseline significance, a 1000-permutation analysis was performed. In each permutation, the Fisher’s exact test was applied on random gene set with the same size (*n* = 195) and expression profile as the imprinted genes. To generate random gene sets with matching expression profile, all genes were first ranked by their median expression level across all datasets and divided into quarters. Random gene sets were accordingly sampled from each quartile in the same proportion as the imprinted genes (Supplementary Text 2). The permutation *P* value was calculated by counting the number of permutations for which there are at least as many datasets with preferential differential expression of imprinted genes (i.e., log_2_ OR > 0).

### Identification of disorder-associated transcriptomic alterations

To identify the genes that are associated with FXS, RTT, DS, and DMD, meta-analysis on the log_2_FC estimates of the differential expression analysis (random effect, inverse variance method, Hartung-Knapp adjustment) was performed using the *meta*^50^ (version 7.0-0) R package. The genes were ranked based on their *P* value. To identify disorder-associated biological processes, the GO-BP terms—clustered by a Resnik similarity threshold of 0.85—were ranked based on the number of significant datasets (GSEA *P* value < 0.05) with a consistent direction of effect (i.e., sign of enrichment score).

### Identification of phenotype-specific transcriptomic alterations

Independent two-group Mann-Whitney U Test was used to find the GO-BP terms that are significantly associated with any of the selected phenotypes (i.e., intellectual disability, hypotonia, global developmental delay, microcephaly, gait ataxia, autism/autistic behavior, seizure, and scoliosis). This was done using both the signed and unsigned -log_10_ *P* values of the GSEA as the dependent variables. Moreover, Fisher’s exact test was applied to identify which genes have a higher likelihood of being differentially expressed (i.e., *P* value < 0.05) for each of selected phenotypes. Protein-protein and gene-gene relationships were retrieved from GeneMANIA^51^ (version 3.5.3).

### Brain region and cell type-specificity

The brain region-specificity of genes of interest was assessed using the six available brain RNA expression datasets from the Human Protein Atlas^11^ (version 24.0): HPA (Human), FANTOM, GTEx, HPA (Pig), HPA (Mouse), and Allen Mouse Brain Atlas. The RNA expression levels for each brain region were expressed in normalized transcript per million values. Only for the Allen Mouse Brain Atlas, the expression energy was used as a measure of RNA expression levels. Moreover, the cell type-specific expression profile of genes of interest was assessed using the processed scRNA-seq data from the Human Brain Atlas^8^ (version 1.0). Finally, the single-cell expression profile of *LHX1* and *LHX5* during the first trimester of the developing human brain was assessed using the processed scRNA-seq data from Braun et al.^10^.

### Shiny app

An online application for the visualization of the collected NDD transcriptomics data was created using the Shiny (version 1.9.1) R package. The application is hosted on the shinyapps.io server and is accessible via the following weblink: https://SyNUM.shinyapps.io/NDD-transcriptomic-atlas/.

### Statistics and reproducibility

All statistical analyses were performed using the R programming language version 4.4.2. The codes used for the analyses are publicly available on GitHub (https://github.com/SyNUM-lab/NDD-transcriptomics) and Zenodo (10.5281/zenodo.15528341).

### Reporting summary

Further information on research design is available in the Nature Portfolio Reporting Summary linked to this article.

## Supplementary information

Transparent Peer Review file

Supplementary Information

Description of Additional Supplementary Materials

Supplementary Data S1

Supplementary Data S2

Supplementary Data S3

Supplementary Data S4

Reporting Summary

## Data Availability

The NDD transcriptomics datasets used in this study are deposited in the GEO. A list of the GEO accession codes is provided in Supplementary Data S1. To facilitate interactive exploration of the transcriptomic profile of the NDD datasets, we have made a Shiny application publicly available at https://SyNUM.shinyapps.io/NDD-transcriptomic-atlas/. Numerical source data for the main figures in the manuscript are available on Zenodo (10.5281/zenodo.15516906).

## Code availability

All R scripts used for the analysis are publicly available on GitHub (https://github.com/SyNUM-lab/NDD-transcriptomics) and Zenodo (10.5281/zenodo.15528341).

## References

[1] Francés, L. et al. Current state of knowledge on the prevalence of neurodevelopmental disorders in childhood according to the DSM-5: a systematic review in accordance with the PRISMA criteria. *Child Adolesc. Psychiatry Ment. Health* **16**, 27 (2022). 35361232 10.1186/s13034-022-00462-1 PMC8973738

[2] Thapar, A., Cooper, M. & Rutter, M. Neurodevelopmental disorders. *Lancet Psychiatry* **4**, 339–346 (2017). 27979720 10.1016/S2215-0366(16)30376-5

[3] D’Souza, H. & Karmiloff-Smith, A. Neurodevelopmental disorders. *Wiley Interdiscip. Rev. Cogn. Sci.* **8**, e1398 (2017). 10.1002/wcs.1398 27906503

[4] Sun, Y. E. & Wu, H. The ups and downs of BDNF in Rett syndrome. *Neuron* **49**, 321–323 (2006). 16446133 10.1016/j.neuron.2006.01.014

[5] Pini, G. et al. IGF1 as a potential treatment for Rett syndrome: safety assessment in six Rett patients. *Autism Res. Treat.* **2012**, 679801 (2012). 22934177 10.1155/2012/679801 PMC3420537

[6] Percy, A. K., Ananth, A. & Neul, J. L. Rett syndrome: the emerging landscape of treatment strategies. *CNS Drugs* 38, 851–867 (2024). 39251501 10.1007/s40263-024-01106-y PMC11486803

[7] Thamban, T., Agarwaal, V. & Khosla, S. Role of genomic imprinting in mammalian development. *J. Biosci.* **45**, 1–21 (2020). 31965998

[8] Siletti, K. et al. Transcriptomic diversity of cell types across the adult human brain. *Science* **382**, eadd7046 (2023). 37824663 10.1126/science.add7046

[9] Gargano, M. A. et al. The human phenotype ontology in 2024: phenotypes around the world. *Nucleic Acids Res.* **52**, D1333–d1346 (2024). 37953324 10.1093/nar/gkad1005 PMC10767975

[10] Braun, E. et al. Comprehensive cell atlas of the first-trimester developing human brain. *Science* **382**, eadf1226 (2023). 37824650 10.1126/science.adf1226

[11] Uhlén, M. et al. Proteomics. Tissue-based map of the human proteome. *Science* **347**, 1260419 (2015). 25613900 10.1126/science.1260419

[12] Isles, A. R. The contribution of imprinted genes to neurodevelopmental and neuropsychiatric disorders. *Transl. Psychiatry* **12**, 210 (2022). 35597773 10.1038/s41398-022-01972-4 PMC9124202

[13] Bahram Sangani, N. et al. Involvement of extracellular vesicle microRNA clusters in developing healthy and Rett syndrome brain organoids. *Cell Mol. Life Sci.* **81**, 410 (2024). 39305343 10.1007/s00018-024-05409-7 PMC11416455

[14] Sharifi, O. et al. Sex-specific single cell-level transcriptomic signatures of Rett syndrome disease progression. *Commun. Biol.* **7**, 1292 (2024). 39384967 10.1038/s42003-024-06990-0 PMC11464704

[15] Wu, H. et al. Genome-wide analysis reveals methyl-CpG-binding protein 2-dependent regulation of microRNAs in a mouse model of Rett syndrome. *Proc. Natl. Acad. Sci. USA* **107**, 18161–18166 (2010). 20921386 10.1073/pnas.1005595107 PMC2964235

[16] Crespi, B. J. & Procyshyn, T. L. Williams syndrome deletions and duplications: genetic windows to understanding anxiety, sociality, autism, and schizophrenia. *Neurosci. Biobehav. Rev.* **79**, 14–26 (2017). 28499504 10.1016/j.neubiorev.2017.05.004

[17] Gutherz, O. R. et al. Potential roles of imprinted genes in the teratogenic effects of alcohol on the placenta, somatic growth, and the developing brain. *Exp. Neurol.* **347**, 113919 (2022). 34752786 10.1016/j.expneurol.2021.113919

[18] Li, J. et al. Potential role of genomic imprinted genes and brain developmental related genes in autism. *BMC Med. Genom.* **13**, 54 (2020). 10.1186/s12920-020-0693-2 PMC7099798 32216802

[19] Michetti, C., Falace, A., Benfenati, F. & Fassio, A. Synaptic genes and neurodevelopmental disorders: From molecular mechanisms to developmental strategies of behavioral testing. *Neurobiol. Dis.* **173**, 105856 (2022). 36070836 10.1016/j.nbd.2022.105856

[20] Nilsberth, C. et al. The ‘Arctic’ APP mutation (E693G) causes Alzheimer’s disease by enhanced Abeta protofibril formation. *Nat. Neurosci.* **4**, 887–893 (2001). 11528419 10.1038/nn0901-887

[21] Xu, W., Tan, L. & Yu, J. T. Link between the SNCA gene and Parkinsonism. *Neurobiol. Aging* **36**, 1505–1518 (2015). 25554495 10.1016/j.neurobiolaging.2014.10.042

[22] Taoufik, E., Kouroupi, G., Zygogianni, O. & Matsas, R. Synaptic dysfunction in neurodegenerative and neurodevelopmental diseases: an overview of induced pluripotent stem-cell-based disease models. *Open Biol.* **8**, 180138 (2018). 10.1098/rsob.180138 PMC6170506 30185603

[23] Ortiz-González, X. R. Mitochondrial dysfunction: a common denominator in neurodevelopmental disorders? *Dev. Neurosci.* **43**, 222–229 (2021). 34350863 10.1159/000517870 PMC8440386

[24] Anitha, A., Thanseem, I., Iype, M. & Thomas, S. V. Mitochondrial dysfunction in cognitive neurodevelopmental disorders: cause or effect? *Mitochondrion* **69**, 18–32 (2023). 36621534 10.1016/j.mito.2023.01.002

[25] Zheng, X. et al. Metabolic reprogramming during neuronal differentiation from aerobic glycolysis to neuronal oxidative phosphorylation. *Elife* **5**, e13374 (2016). 10.7554/eLife.13374 PMC4963198 27282387

[26] van Horssen, J., van Schaik, P. & Witte, M. Inflammation and mitochondrial dysfunction: a vicious circle in neurodegenerative disorders? *Neurosci. Lett.* **710**, 132931 (2019). 28668382 10.1016/j.neulet.2017.06.050

[27] Zengeler, K. E. & Lukens, J. R. Innate immunity at the crossroads of healthy brain maturation and neurodevelopmental disorders. *Nat. Rev. Immunol.* **21**, 454–468 (2021). 33479477 10.1038/s41577-020-00487-7 PMC9213174

[28] Hickman, R. A., O’Shea, S. A., Mehler, M. F. & Chung, W. K. Neurogenetic disorders across the lifespan: from aberrant development to degeneration. *Nat. Rev. Neurol.* **18**, 117–124 (2022). 34987232 10.1038/s41582-021-00595-5 PMC10132523

[29] Fortea, J. et al. Clinical and biomarker changes of Alzheimer’s disease in adults with down syndrome: a cross-sectional study. *Lancet* **395**, 1988–1997 (2020). 32593336 10.1016/S0140-6736(20)30689-9 PMC7322523

[30] Utari, A. et al. Aging in fragile X syndrome. *J. Neurodev. Disord.* **2**, 70–76 (2010). 20585378 10.1007/s11689-010-9047-2 PMC2882562

[31] Zhang, W. et al. Astrocytes increase exosomal secretion of oligodendrocyte precursor cells to promote their proliferation via integrin β4-mediated cell adhesion. *Biochem. Biophys. Res. Commun.* **526**, 341–348 (2020). 32220495 10.1016/j.bbrc.2020.03.092

[32] Bahram Sangani, N., Gomes, A. R., Curfs, L. M. G. & Reutelingsperger, C. P. The role of extracellular vesicles during CNS development. *Prog. Neurobiol.* **205**, 102124 (2021). 34314775 10.1016/j.pneurobio.2021.102124

[33] Nguyen, M. V. et al. Oligodendrocyte lineage cells contribute unique features to Rett syndrome neuropathology. *J. Neurosci.* **33**, 18764–18774 (2013). 24285883 10.1523/JNEUROSCI.2657-13.2013 PMC3841446

[34] Duan, D., Goemans, N., Takeda, S., Mercuri, E. & Aartsma-Rus, A. Duchenne muscular dystrophy. *Nat. Rev. Dis. Prim.* **7**, 13 (2021). 33602943 10.1038/s41572-021-00248-3 PMC10557455

[35] Turczyńska, K. M. et al. Regulation of smooth muscle dystrophin and synaptopodin 2 expression by actin polymerization and vascular injury. *Arterioscler Thromb. Vasc. Biol.* **35**, 1489–1497 (2015). 25857312 10.1161/ATVBAHA.114.305065

[36] Gunst, S. J. & Zhang, W. Actin cytoskeletal dynamics in smooth muscle: a new paradigm for the regulation of smooth muscle contraction. *Am. J. Physiol. Cell Physiol.* **295**, C576–C587 (2008). 18596210 10.1152/ajpcell.00253.2008 PMC2544441

[37] Murk, K., Ornaghi, M. & Schiweck, J. Profilin isoforms in health and disease - all the same but different. *Front. Cell Dev. Biol.* **9**, 681122 (2021). 34458253 10.3389/fcell.2021.681122 PMC8387879

[38] Bianchi, R. et al. Hippocampal synaptic and membrane function in the DBA/2J-mdx mouse model of Duchenne muscular dystrophy. *Mol. Cell Neurosci.* **104**, 103482 (2020). 32171922 10.1016/j.mcn.2020.103482

[39] Zhao, Y. et al. LIM-homeodomain proteins Lhx1 and Lhx5, and their cofactor Ldb1, control Purkinje cell differentiation in the developing cerebellum. *Proc. Natl. Acad. Sci. USA* **104**, 13182–13186 (2007). 17664423 10.1073/pnas.0705464104 PMC1941824

[40] Lui, N. C. et al. Lhx1/5 control dendritogenesis and spine morphogenesis of Purkinje cells via regulation of Espin. *Nat. Commun.* **8**, 15079 (2017). 28516904 10.1038/ncomms15079 PMC5454373

[41] Ming, X., Prasad, N., Thulasi, V., Elkins, K. & Shivamurthy, V. K. N. Possible contribution of cerebellar disinhibition in epilepsy. *Epilepsy Behav.* **118**, 107944 (2021). 33887658 10.1016/j.yebeh.2021.107944

[42] Bernardi, S., Gemignani, F. & Marchese, M. The involvement of Purkinje cells in progressive myoclonic epilepsy: focus on neuronal ceroid lipofuscinosis. *Neurobiol. Dis.* **185**, 106258 (2023). 37573956 10.1016/j.nbd.2023.106258 PMC10480493

[43] Cook, A. A., Fields, E. & Watt, A. J. Losing the beat: contribution of Purkinje cell firing dysfunction to disease, and its reversal. *Neuroscience* **462**, 247–261 (2021). 32554108 10.1016/j.neuroscience.2020.06.008

[44] Leung, R. F. et al. Genetic regulation of vertebrate forebrain development by homeobox genes. *Front. Neurosci.* **16**, 843794 (2022). 35546872 10.3389/fnins.2022.843794 PMC9081933

[45] Hirsch, D., Kohl, A., Wang, Y. & Sela-Donenfeld, D. Axonal projection patterns of the dorsal interneuron populations in the embryonic hindbrain. *Front. Neuroanat.* **15**, 793161 (2021). 35002640 10.3389/fnana.2021.793161 PMC8738170

[46] Barrett, T. et al. NCBI GEO: archive for functional genomics data sets-update. *Nucleic Acids Res.* **41**, D991–D995 (2013). 23193258 10.1093/nar/gks1193 PMC3531084

[47] Love, M. I., Huber, W. & Anders, S. Moderated estimation of fold change and dispersion for RNA-seq data with DESeq2. *Genome Biol.* **15**, 550 (2014). 25516281 10.1186/s13059-014-0550-8 PMC4302049

[48] Xu, S. et al. Using clusterProfiler to characterize multiomics data. *Nat. Protoc.* 19, 3292–3320 (2024). 39019974 10.1038/s41596-024-01020-z

[49] Sayols, S. Rrvgo: a bioconductor package for interpreting lists of gene ontology terms. *MicroPubl. Biol.* **10**, 000811 (2023). 10.17912/micropub.biology.000811 PMC10155054 37151216

[50] Balduzzi, S., Rücker, G. & Schwarzer, G. How to perform a meta-analysis with R: a practical tutorial. *Evid. Based Ment. Health* **22**, 153–160 (2019). 31563865 10.1136/ebmental-2019-300117 PMC10231495

[51] Warde-Farley, D. et al. The GeneMANIA prediction server: biological network integration for gene prioritization and predicting gene function. *Nucleic Acids Res.* **38**, W214–W220 (2010). 20576703 10.1093/nar/gkq537 PMC2896186

