# Supplementary material for: Integrative analysis of 115 transcriptomic studies decodes the molecular landscape of neurodevelopmental disorders
Source: Commun Biol. 2025 Jun 12;8:914. doi: 10.1038/s42003-025-08330-2 (PMC12159135; doi:10.1038/s42003-025-08330-2)
Supplement: Supplementary file 2 — Supplementary Information [file 42003_2025_8330_MOESM2_ESM.pdf]

# Supplementary Information

## Integrative analysis of 115 transcriptomic studies decodes the molecular landscape of neurodevelopmental disorders

Jarno Koetsier, Lars M.T. Eijssen, Leon J. Schurgers, Leopold M.G. Curfs, Chris P. Reutelingsperger, Nasim Bahram Sangani

**Supplementary Text S1** GEO search query

*("lesch-nyhan syndrome"[All Fields] OR "lowe syndrome"[All Fields] OR "rubinstein-taybi syndrome"[All Fields] OR "cornelia de lange syndrome"[All Fields] OR "cri du chat syndrome"[All Fields] OR "galactosaemia"[All Fields] OR "angelman syndrome"[All Fields] OR "williams syndrome"[All Fields] OR "marfan syndrome"[All Fields] OR "prader-willi syndrome"[All Fields] OR "rett syndrome"[All Fields] OR "phenylketonuria"[All Fields] OR "duchenne muscular dystrophy"[All Fields] OR "tuberous sclerosis"[All Fields] OR "trisomy 18"[All Fields] OR "velocardiofacial syndrome"[All Fields] OR "neurofibromatosis type 1"[All Fields] OR "turner syndrome"[All Fields] OR "xyy"[All Fields] OR "xxx"[All Fields] OR "noonan syndrome"[All Fields] OR "fragile x syndrome"[All Fields] OR "klinefelter syndrome"[All Fields] OR "fetal alcohol syndrome"[All Fields] OR "cerebral palsy"[All Fields] OR "down syndrome"[All Fields] OR "tourette syndrome"[All Fields] OR "autism spectrum disorder"[All Fields] OR "developmental dyscalculia"[All Fields] OR "attention deficit hyperactivity disorder"[All Fields] OR "intellectual disability"[All Fields] OR "developmental dyslexia"[All Fields] OR "developmental coordination disorder"[All Fields] OR "specific language impairment"[All Fields] OR "speech sound disorder") AND "GEO2R"[All Fields] AND "Homo sapiens"[porgn] AND "Expression profiling by high throughput sequencing"[Filter]*

**Supplementary Text S2** Pseudo-code of permutation analysis

1. *For each gene, calculate the median gene expression value across all datasets.*
2. *Sort the genes based on their median expression value and divide them into quarters: Q1, Q2, Q3, Q4. Here, Q1 and Q4 include the genes with the highest and lowest median expression value, respectively.*
3. *Get the number of imprinted genes per quarter.*
4. *For each permutation, randomly sample each quarter in the same proportion as imprinted genes. The random sample is the same size as the number of imprinted genes.*
5. *For each random gene set, calculate the odds ratio of differential expression per dataset (Fisher's exact test).*

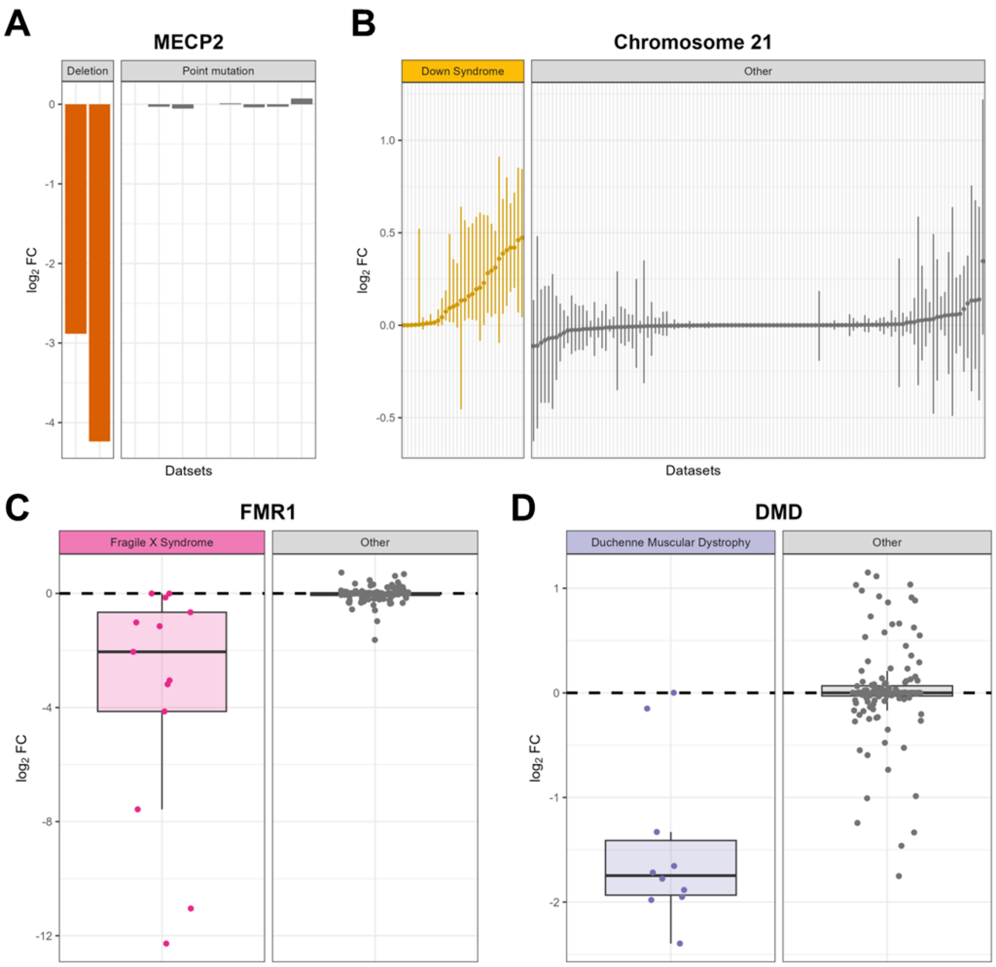


**Supplementary Figure S1.** Expression profile of the causative genes of Rett Syndrome, Down Syndrome, Fragile X Syndrome, and Duchenne muscular dystrophy. **A)** *MECP2* is strongly downregulated in Rett syndrome datasets with (partial) *MECP2* deletion, while its expression remains unchanged in the Rett syndrome datasets with a *MECP2* point mutation. **B)** The interquartile ranges of chromosome 21 genes in Down syndrome and the other datasets. Chromosome 21 genes are upregulated in most Down syndrome datasets, while there is no consistent upregulation of these genes in other neurodevelopmental disorders. **C)** *FMR1* expression is strongly downregulated in Fragile X syndrome datasets. **D)** *DMD* expression is consistently downregulated in the Duchenne muscular dystrophy datasets.

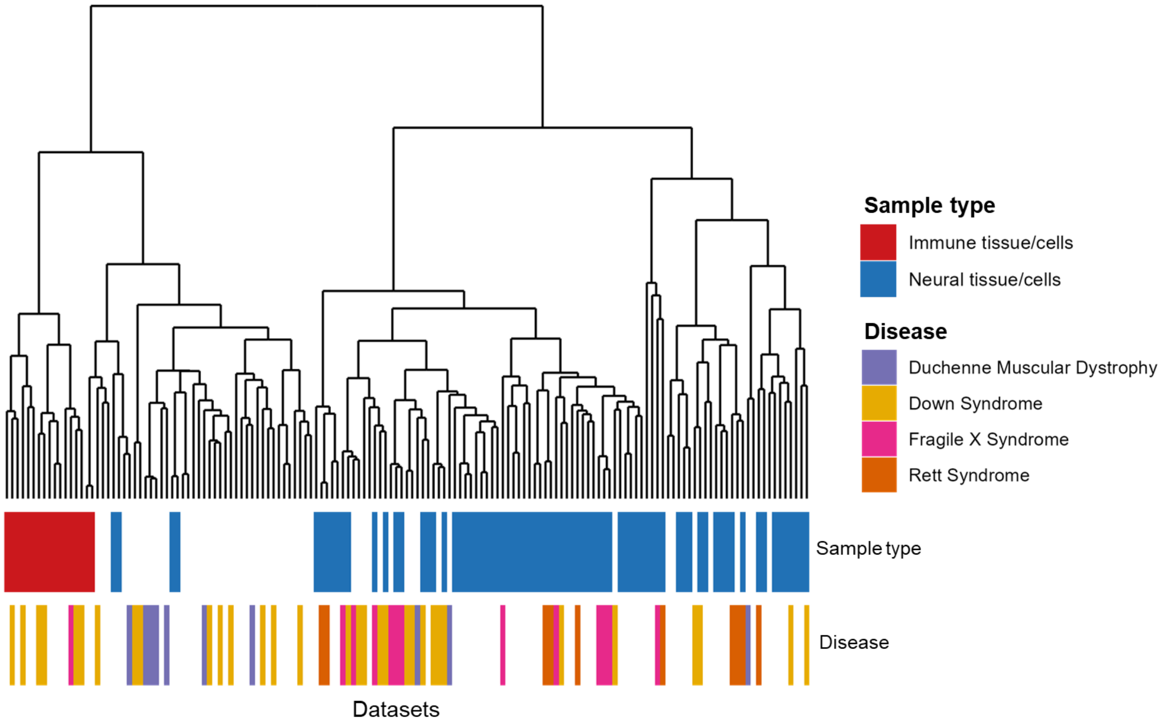


**Supplementary Figure S2.** Dendrogram showing the similarity between the 151 datasets. The distance between the datasets was calculated using the Spearman correlation of the gene's P values. The gene's P values were calculated for each dataset through the differential expression analysis of neurodevelopmental disorders cases versus controls. Ward D2 was used as linkage method.

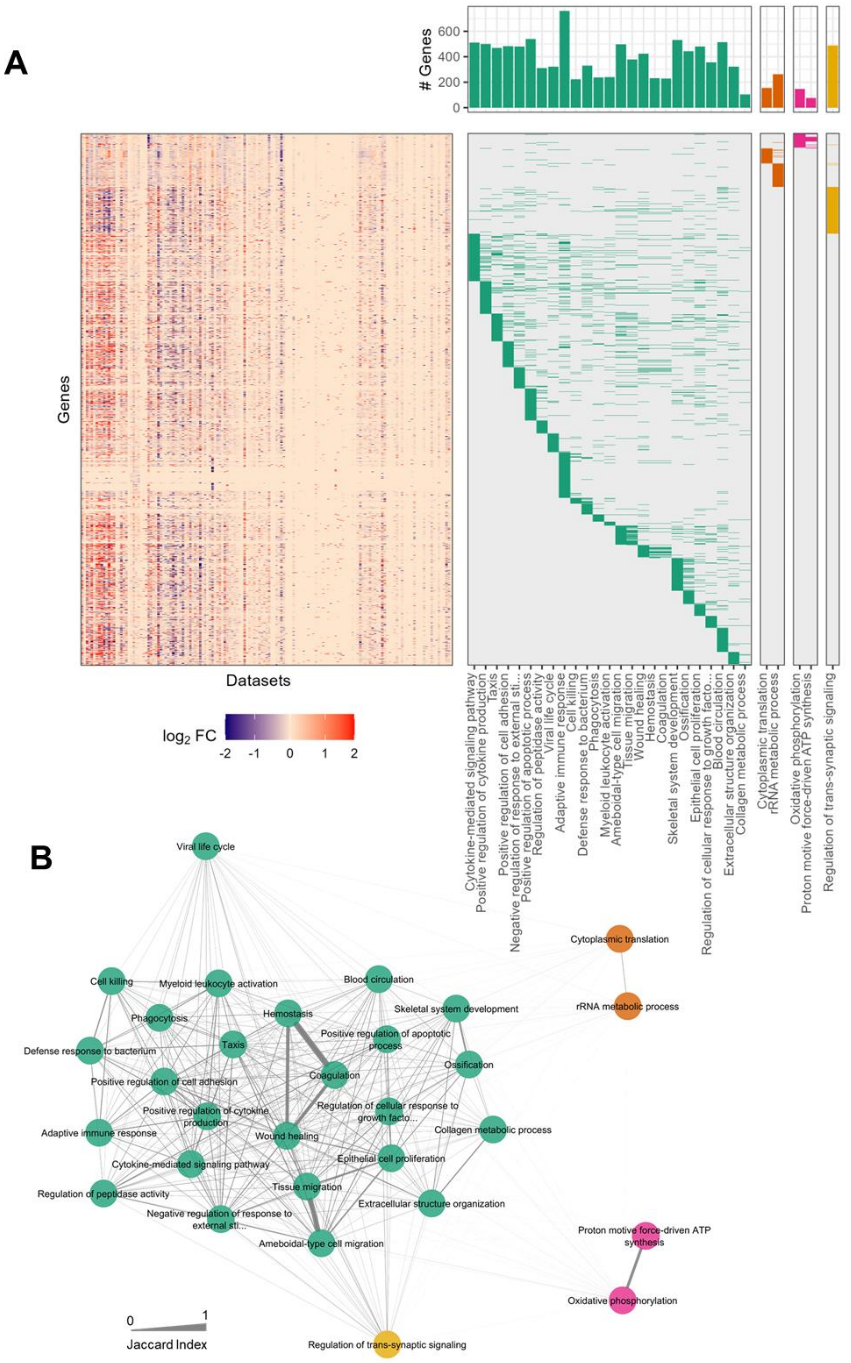


**Supplementary Figure S3.** Overview of the 30 GO-BP terms that were identified to exhibit transcriptomic changes across the NDD spectrum (see Figure 2 in manuscript). **A)** Heatmap of the $\log_2$FCs for the genes in the 30 GO-BP terms across the 151 datasets (left panel). The right panel shows how many genes are associated to each GO-BP term (top) and to which GO-BP term(s) each gene is associated (bottom). An overview of which genes are included in each GO term is provided in Supplementary Dataset S2. **B)** Network diagram showing the similarity between the 30 GO-BP terms. The similarity (edge width) between two GO-BP terms is expressed as the Jaccard Index (*i.e.*, size of the intersection divided by the size of the union).

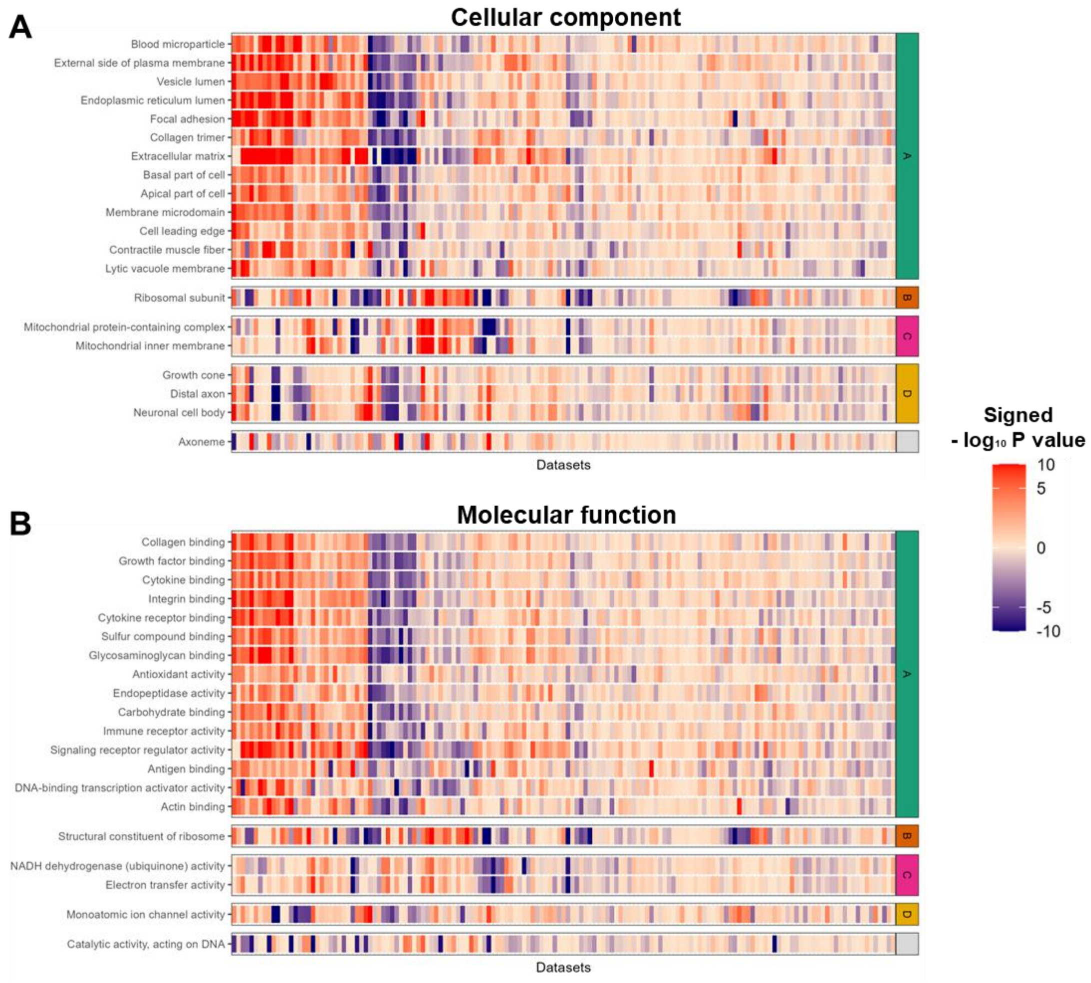


**Supplementary Figure S4.** Common alterations between the neurodevelopmental disorders (NDDs). **A)** Heatmap of the enrichment of the top 20 most frequently enriched Gene Ontology – Cellular component (GO-CC) terms across the 151 NDD datasets. **B)** Heatmap of the enrichment of the top 20 most frequently enriched Gene Ontology – Molecular function (GO-MF) terms across the 151 NDD datasets.

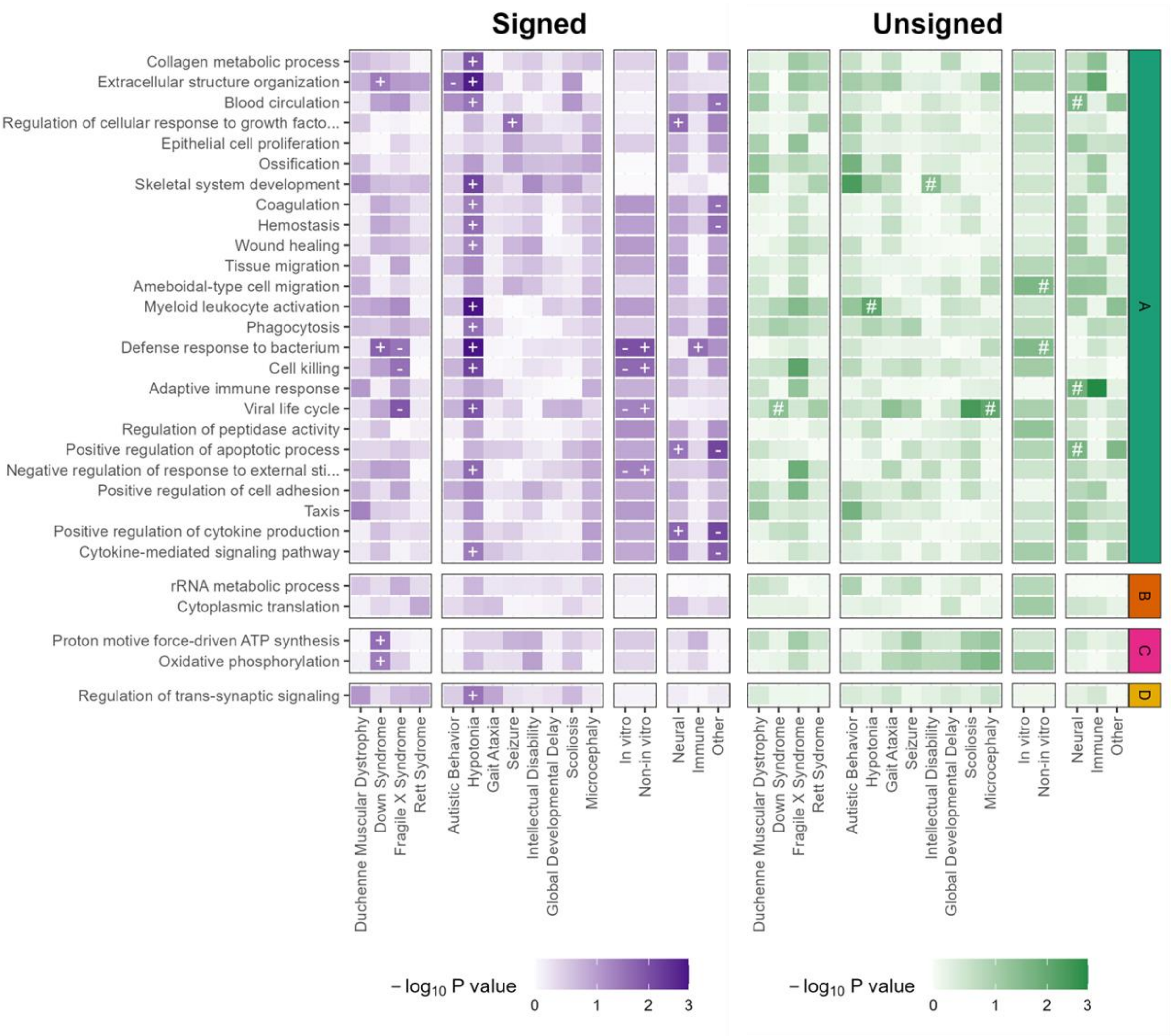


**Supplementary Figure S5.** Association of the top 30 GO-BP terms with specific neurodevelopmental disorders, neurological phenotypes, model systems, and sample types. Independent two-group Mann-Whitney U Test was used to find the GO-BP terms (*i.e.*, signed and unsigned -$\log_{10}$ P value) that are significantly associated with any of the four neurodevelopmental disorders *(i.e.*, Duchenne muscular dystrophy, Down Syndrome, Fragile X Syndrome, Rett Syndrome), eight neurological phenotypes (*i.e.*, intellectual disability, hypotonia, global developmental delay, microcephaly, gait ataxia, autism/autistic behavior, seizure, and scoliosis), (non)-*in vitro* model systems, and sample types (*i.e.,* immune, neural, and other cell types/tissues). After FDR-adjustment, none of the GO-BP terms correlate with any of the phenotypes, disorders, model systems, or sample types. In the left panel, nominally significantly higher levels of positive enrichment (positive -$\log_{10}$ P value) in the associated datasets are indicated by "+", while nominally significantly higher levels of negative enrichment (negative -$\log_{10}$ P value) in the associated datasets are indicated by "-". In the right panel, nominally significantly higher levels of enrichment (unsigned -$\log_{10}$ P value) in the associated datasets are indicated by "#". P value < 0.05 is considered nominally significant.

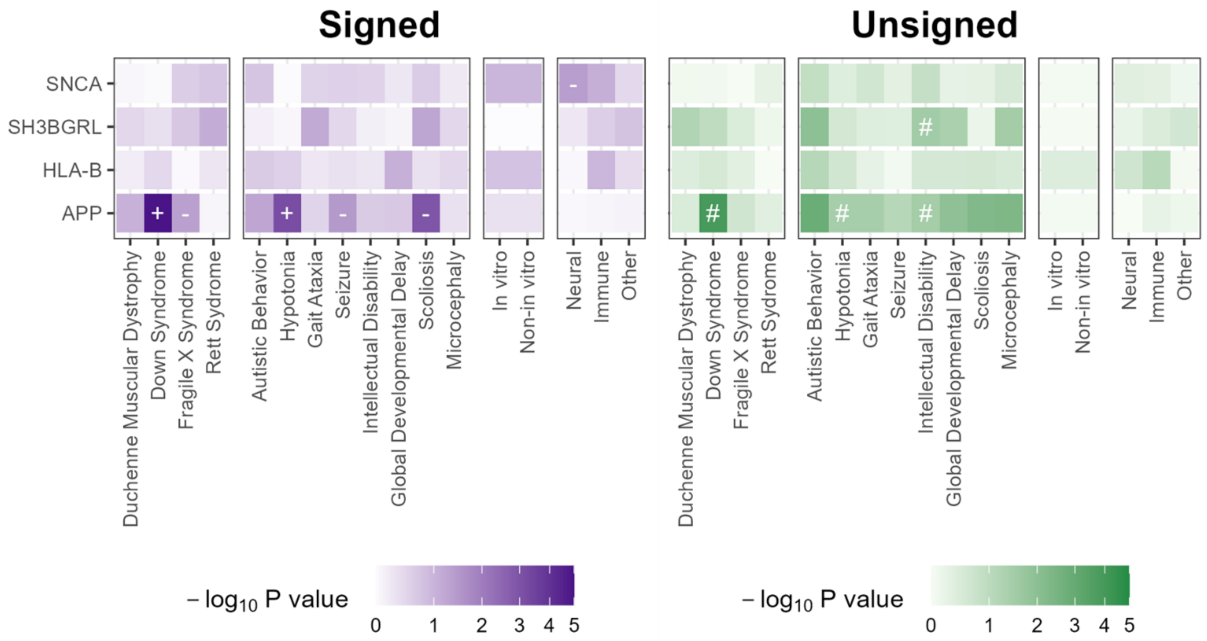


**Supplementary Figure S6.** Association of *SNCA*, *SH3BGRL*, *HLA-B*, and *APP* with neurodevelopmental disorders, neurological phenotypes, model systems, and sample types. Independent two-group Mann-Whitney U Test was used to identify whether the $log_2FC$ (signed) and absolute $log_2FC$ (unsigned) profile of these four genes are significantly associated with any of the four neurodevelopmental disorders *(i.e.*, Duchenne muscular dystrophy, Down Syndrome, Fragile X Syndrome, Rett Syndrome), eight neurological phenotypes (*i.e.*, intellectual disability, hypotonia, global developmental delay, microcephaly, gait ataxia, autism/autistic behavior, seizure, and scoliosis), (non)-*in vitro* model systems, and sample types (*i.e.,* immune, neural, and other cell types/tissues). Because of its location on chromosome 21, *APP* exhibits higher $log_2FCs$ in Down syndrome than in any other disease (independent two-group Mann-Whitney U Test, FDR-adj. P value = 3.9e-7). After FDR-adjustment, the $log_2FC$ profiles of *HLA-B*, *SH3BGRL*, and *SCNA* are not associated with any specific disorder, phenotype, sample type, or model system (independent two-group Mann-Whitney U Test, FDR-adj. P value > 0.05). In the left panel, nominally significantly higher $log_2FCs$ in the associated datasets are indicated by “+”, while nominally significantly lower $log_2FCs$ in the associated datasets are indicated by “-“. In the right panel, nominally significantly higher absolute $log_2FCs$ in the associated datasets are indicated by “#”. P value < 0.05 is considered nominally significant.

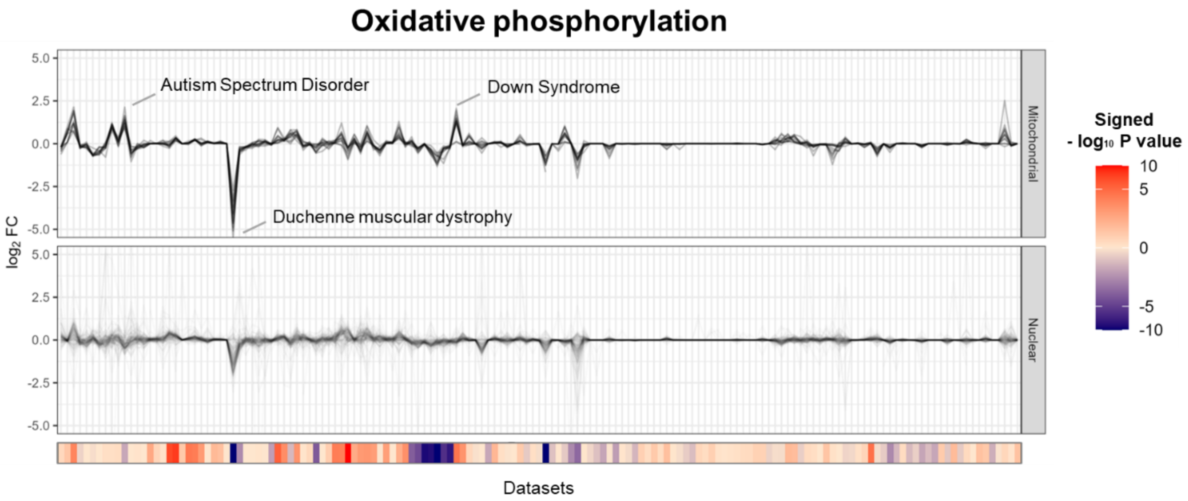


**Supplementary Figure S7.** $log_2FC$ profile of mitochondrial- and nuclear-encoded genes involved in oxidative phosphorylation across the 151 NDD datasets. The bottom color bar shows the signed $-log_{10}$ P value of the Gene Set Enrichment Analysis (GSEA) of the *Oxidative phosphorylation* (GO:0006119) term. Overall, mitochondrial-encoded genes show stronger transcriptomic alterations than nuclear-encoded genes.

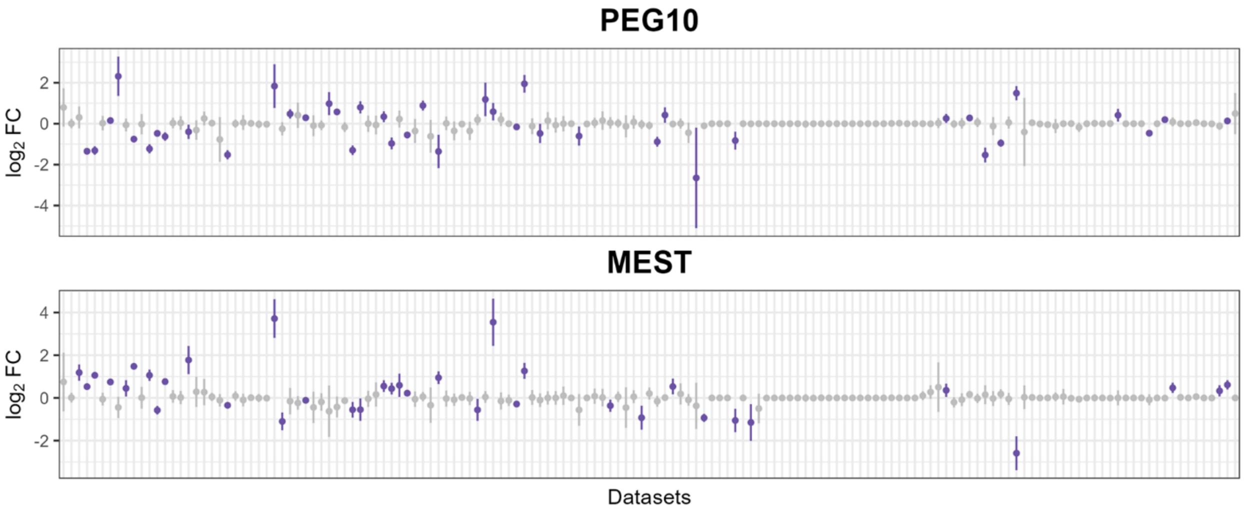


**Supplementary Figure S8.** $\log_2$FC profile of the imprinted genes *PEG10* and *MEST*. Colored data points are statistically significant (P value < 0.05).

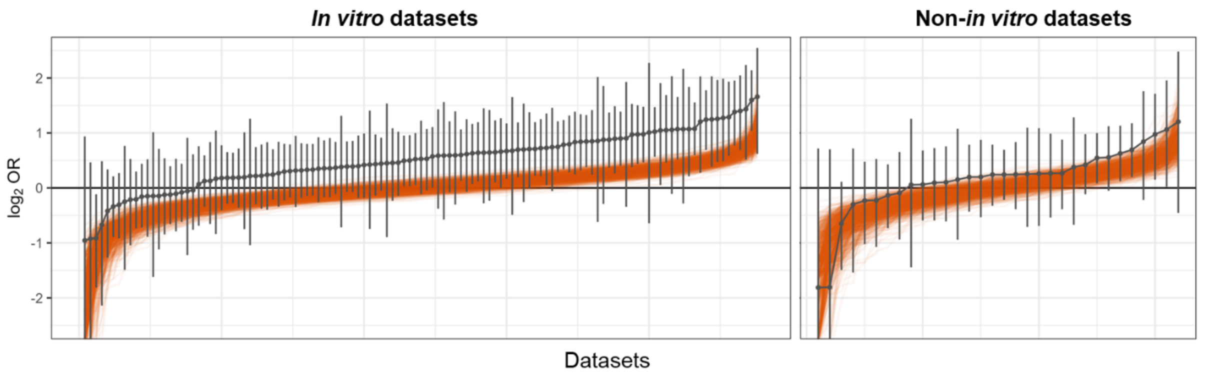


**Supplementary Figure S9.** The $\log_2$ Odds Ratio ($\log_2$ OR) and the 95% confidence interval (C.I) of differential expression (P value < 0.05) of imprinted versus non-imprinted genes. Imprinted genes have higher odds of being differentially expressed than non-imprinted genes ($\log_2$ OR > 0) in 99 of the 119 *in vitro* datasets (*e.g.,* stem cell-derived, fibroblast, and immortalized cell lines) and 24 of the 32 non-*in vitro* datasets (*e.g.,* blood, biopsy, and post-mortem tissues) datasets. The orange lines show the ORs of 1000 random gene sets with the same size and expression profile as the imprinted genes. The higher odds of differential expression of imprinted genes occurs more than expected by chance both in *in vitro* (permutation P value < 0.001) and non-*in vitro* (permutation P value = 0.003) datasets.

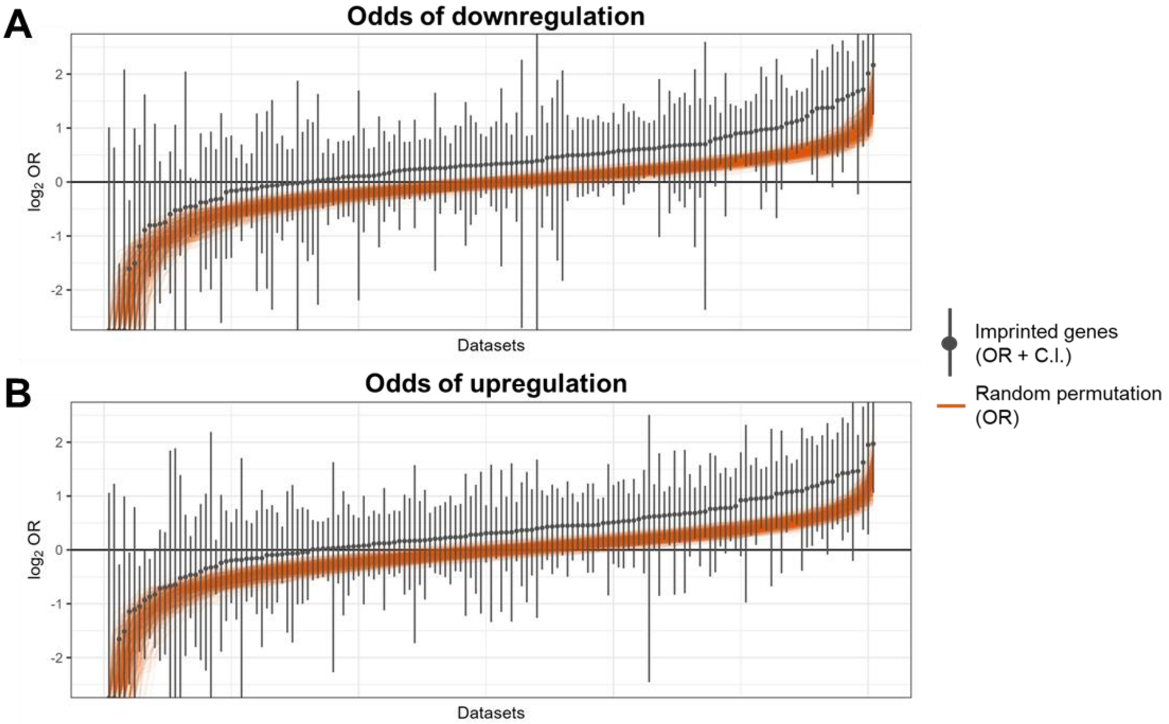


**Supplementary Figure S10.** Odds of up- and downregulation of imprinted genes. **A)** The $\log_2$ Odds Ratio (OR) and the 95% confidence interval (C.I) of being <u>downregulated</u> (P value < 0.05 and logFC < 0) of imprinted versus non-imprinted genes. The orange lines show the ORs of 1000 random gene sets with the same size and expression profile as the imprinted genes. This shows that the odds of imprinted genes to be differentially expressed is significantly higher than expected by chance. **B)** Same as panel A, but instead the $\log_2$ OR and the C.I. of being <u>upregulated</u> (P value < 0.05 and logFC > 0) of imprinted versus non-imprinted genes is shown.

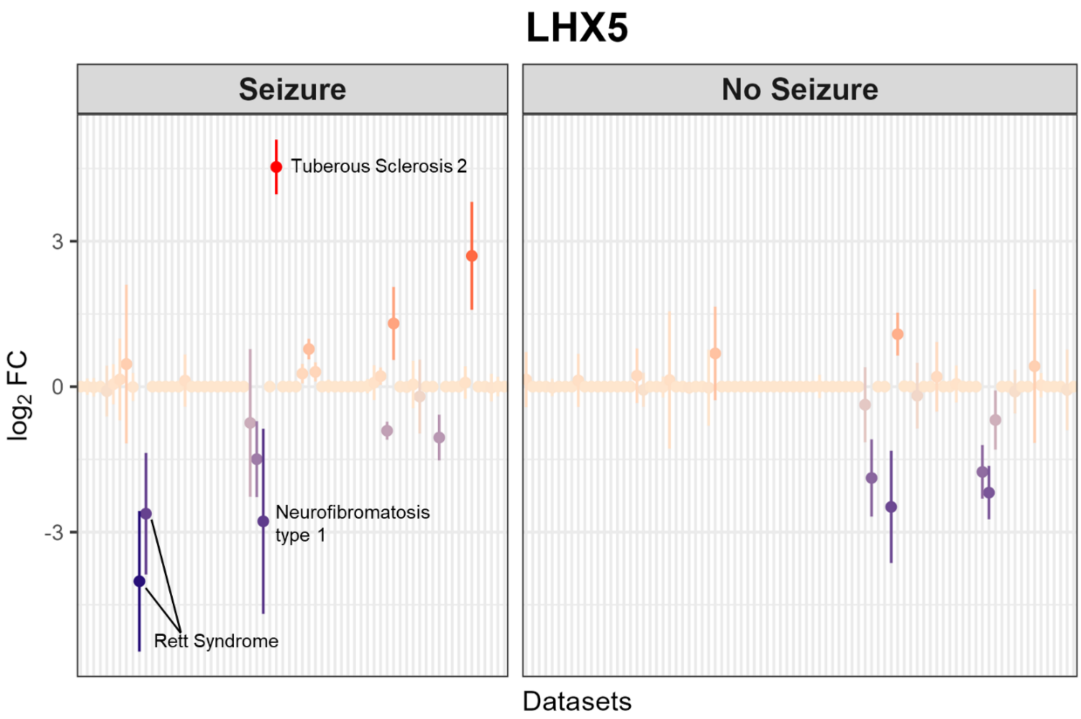


**Supplementary Figure S11.** $\log_2$ Fold Change (FC) profile and their 95% confidence interval of the *LHX5* gene in seizure- and non-seizure-associated neurodevelopmental disorders

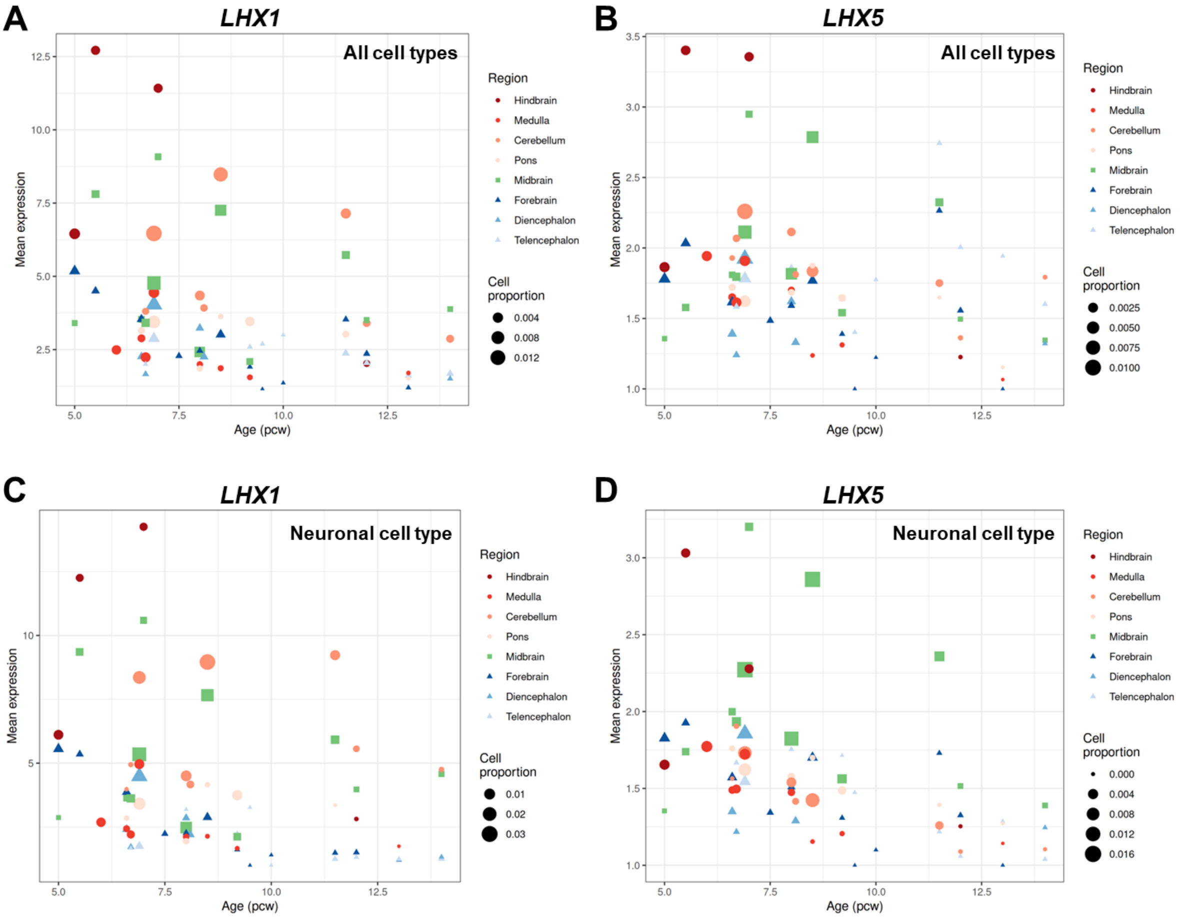


**Supplementary Figure S12**. Expression of *LHX1* and *LHX5* in first trimester developing human brain. The processed single cell RNA expression and annotations from Braun *et al.*[1] were downloaded from CZ CELLxGENE Discover (https://cellxgene.cziscience.com/collections/4d8fed08-2d6d-4692-b5ea-464f1d072077). The age in postconceptional weeks (pcw) is plotted against the mean expression of *LHX1* and *LHX5* in the expressing cell fraction. The expressing cell fraction, as indicated by the point size, is the proportion of cells with a read count of at least one. **A)** and **B)** show the expression of *LHX1* and *LHX5* in all cell types combined, while **C)** and **D)** show the expression of *LHX1* and *LHX5* in neurons.

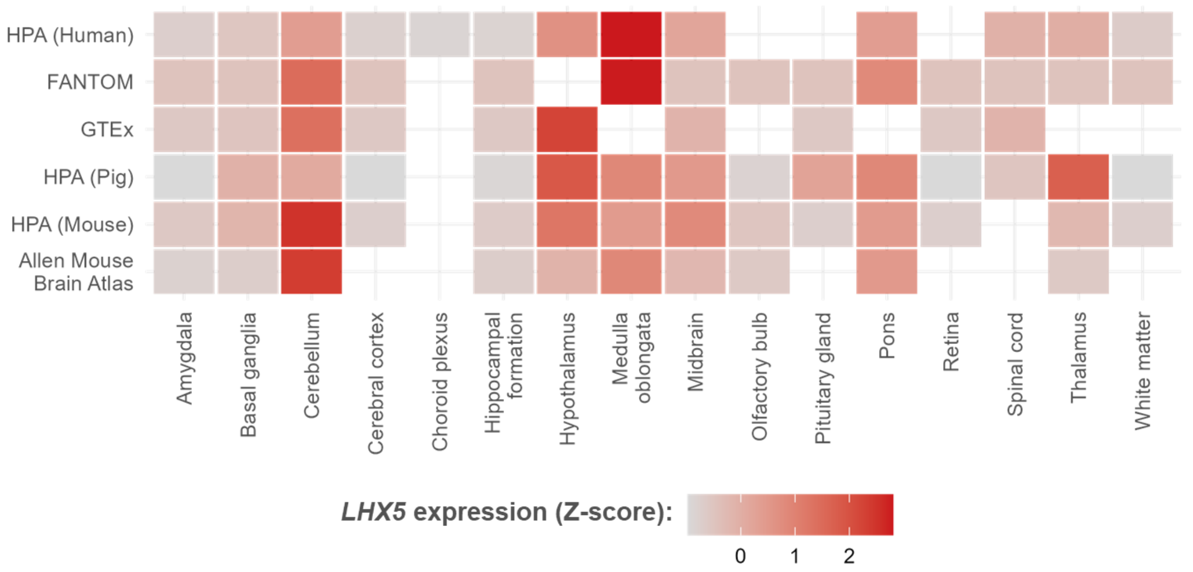


**Supplementary Figure S13.** *LHX5* expression levels across different brain regions in six different datasets available from the Human Protein Atlas: HPA (Human), FANTOM, GTEx, HPA (Pig), HPA (Mouse), and Allen Mouse Brain Atlas. The Z-score was calculated for each dataset separately.

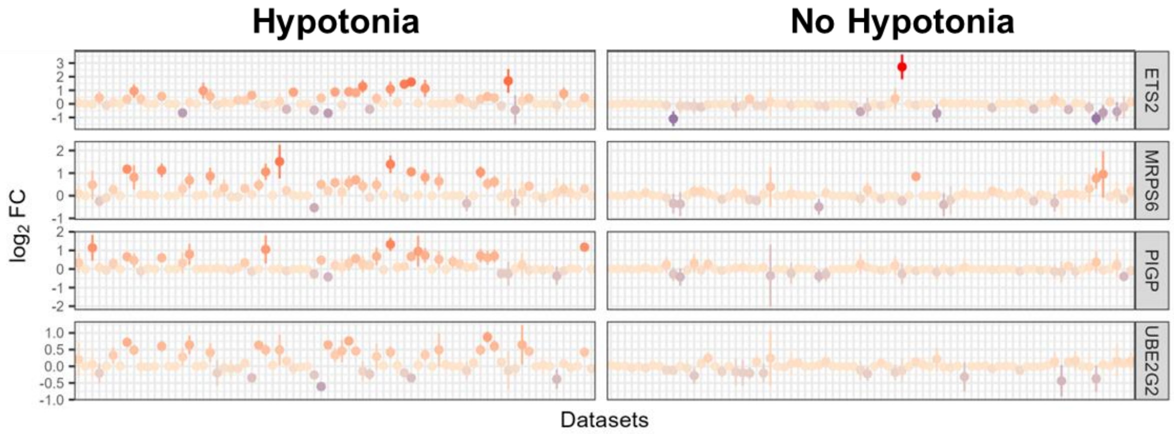


**Supplementary Figure S14.** $log_2FC$ profile of *EST2*, *MRPS6*, *PIGP*, and *UBE2G2* in hypotonia-associated neurodevelopmental disorders.

## Supplementary References

1 Braun, E. *et al.* Comprehensive cell atlas of the first-trimester developing human brain. *Science* **382**, eadf1226 (2023). https://doi.org/10.1126/science.adf1226
