# Supplementary material for: Integrative analysis of 115 transcriptomic studies decodes the molecular landscape of neurodevelopmental disorders
Source: Commun Biol. 2025 Jun 12;8:914. doi: 10.1038/s42003-025-08330-2 (PMC12159135; doi:10.1038/s42003-025-08330-2)
Supplement: Supplementary file 3 — Description of Additional Supplementary Materials [file 42003_2025_8330_MOESM3_ESM.pdf]

Description of Additional Supplementary Files

File name: Supplementary Data 1

Description: Overview of the included RNA-seq datasets.

File name: Supplementary Data 2

Description: Overview of the genes and their log2FCs in the 30 GO-BP terms with a differential expression profile across the NDD datasets.

File name: Supplementary Data 3

Description: Statistics of the transcriptome-wide meta-analysis for DMD, DS, FXS, and RTT.

File name: Supplementary Data 4

Description: Overview of the genes that were found to be more likely upregulated in hypotonia-associated NDDs than in NDDs without hypotonia.
