# Supplementary material for: Integrative analysis of 115 transcriptomic studies decodes the molecular landscape of neurodevelopmental disorders
Source: Commun Biol. 2025 Jun 12;8:914. doi: 10.1038/s42003-025-08330-2 (PMC12159135; doi:10.1038/s42003-025-08330-2)
Supplement: Supplementary file 1 — Transparent Peer Review file [file 42003_2025_8330_MOESM1_ESM.pdf]

nature portfolio

Peer Review File

Integrative analysis of 115 transcriptomic studies decodes the molecular landscape of neurodevelopmental disorders

Corresponding Author: Mr Jarno Koetsier

This file contains all reviewer reports in order by version, followed by all author rebuttals in order by version.

Version 0:

Reviewer comments:

Reviewer #1

(Remarks to the Author)
"Integrative analysis of 115 transcriptomic studies decodes the molecular landscape of neurodevelopmental disorders"

The analysis presented in this study is very original and relevant. The article is well-structured and the conclusions of this study are very relevant for the analysis of the implications of genetic alterations at the transciptomic level in NDDs. However, I think that some aspects of the analysis can be improved, here are my suggestions:

Graphical abstract
- As the authors decided to include a graphical abstract, I suggest that the authors improve the detail of the information provided, as for instance, the second part of the image is not informative, particullarly what the authors mean with “common alterations”.

Methods/Results
- Can you supply a supplementary table demonstrating that the datasets cluster better by cell type than by disease, as this is a very interesting observation and a key result of your analysis, and I think that the data shown in figure 1D is not sufficient to illustrate this conclusion.
- The authors perform Gene Set Enrichment Analysis on the 151 datasets to find Gene Ontology Biological Process terms with a differential expression profile across the NDDs. The 30 GO-BP terms that reached statistical significance were shown and were gouped in 4 different subgroups; However, you don’t explain how you perform this subgrouping. Additionally, I would like that you add a table showing which are the genes implicated in the 30 GO terms, also because it is important to have a picture of how many genes define your 4 groups of biological processes and the level of signifficance, to understand how genes are contributing to define BPs. It is not clear to me from the data shown that the gene APP that you refer as being largely altered across several datasets is not related with sinaptyc signalling as well as SCNA, since APP function is associated to neuronal migration. I wonder if you have enough evidence to descriminate between the groups 3 and 4, as it is possible that the GO-BPs associated to mitochondrial function are related with neuronal development. I here suggest that you also compute the GO molecular functions and GO celular components to clarify this question, and the interpretation and discussion of the subgrouping presented by the authors.

Discussion
- The identification of disease-specific transcriptomic changes for DMD, DS, FXS, and RTT is quite interesting since these disorders are usually mainly discussed in the context of single-gene mutations. In this context, add some lines in the discussion on how your analysis adds to previous evidence of gene association to such monogenic syndromic disorders.
- As the authors found that differential expression of cerebellar Purkinje cell layer formation was strongly correlated with seizure-associated NDDs, I suggest adding some sentences on the importance of the GABAergic signalling involving cerebellar Purkinje cells and about the evidence linking this pathway with inhibitory/excitatory imbalance, seizures and disease (for a review in this subject: https://www.sciencedirect.com/science/article/pii/S0969996123002735). The focus of this final discussion can be shifted towards the implications of the abnormal cerebellar Purkinje cell layer formation to the the inhibitory signalling, and you can highlight that your analysis suggests an alternative mechanism for this dysregulation, other than the dysregulation of neurotransmission or receptor pathways, here involving cerebellar Purkinje cell layer formation (-as the authors also mention) and probably result from differential gene expression from the genes LHX1 and LHX5.

Reviewer #2

(Remarks to the Author)
The manuscript describes a large study of neurodevelopmental disorders (NDDs) RNA-seq datasets. The aim of this study is to gain power and take a cross-disorder approach in order to gain insight into general mechanisms that might underpin neurodevelopmental disorders broadly. I think the approach is interesting and understandable, given the problems with power seen in RNA-seq studies sometimes. This approach also yields some interesting patterns. However, although I have no problem with the approach, there are some important details that are missing which makes the interpretability of these findings less clear.
Firstly, the authors highlight significantly increased changes in imprinted gene expression than expected by chance. I really like the fact the authors have focused on these genes and the rationale for this is robust. However, I could not find the list of imprinted genes used in this study. There are many contrasting ideas as to the extent of genomic imprinting, with some using differential transcriptomic studies to suggest there are >2000. However, more conservative (and probably accurate) estimates indicate ~250 in humans.The fact that the authors have a comparable list of 195 suggests they have used conservative lists like others more recently (10.1186/s12864-022-08986-8). But how this list was generated is not apparent.
Another issue is highlighted by the fact that one of the key findings was that ageing-related transcriptomic changes were a significant group inthe analysis across NDDs. This is interesting but, as the authors do mention, is this driven by the large representation of Downs Syndrome (DS) samples, which represent >20% of all the samples analysed? We know that DS have accelerated ageing and often develop early onset Alzheimer's disease - could there over-representation be a factor for this finding more generally? This raises the question of whether there was any weighting given to normalise the contribution of the four highly represented disorders (RTT, DS, FXS and DMD)? This isn't clear from the analysis methods, but may of course be something to consider at least.
Finally, although the details of the sources (i.e tissue) of the RNA-seq data are available in the supplementary material, I think these need some discussion in the main text. A key factor for me when mining these data is that most are not tissue samples at all, but are in fact in vitro derived lines - 91/151 (~60%) were iPSC / ESC / cell line -derived samples. I appreciate that this is not unexpected, but it could be clearer. Moreover, how much of an impact does this have on the findings? For instance, there is a literature indicating that imprinted gene expression is not 100% faithful in in vitro systems - how much is this driving the changes in imprinted genes seen here? Similarly, only 56 of the datasets are on CNS sample-types (Neuron / Brain / NPC) - how does this affect the patterns (and relevance) of GO-terms identified? The slightly disappointing thing is that this could have been explored here, as there is possibly the Power to analyse subsets of these data to test these questions. For instance, are the patterns of change broadly similar if all in vitro system samples are excluded?

Reviewer #3

(Remarks to the Author)
I enjoyed reading the paper. Beautiful figures, clear and straightforward writing style, and the analysis seems sound. I also appreciate that the authors created a database for exploring their analysis. However, the findings do not seem very novel. The authors state themselves that imprinted genes, synaptic signaling, mitochondrial processes, and inflammatory responses are already associated with NDDs. I also do not find wholly convincing that their results point to a connection with neurodegenerative disease. Will not diseases with very different pathologies anyway converge on dysregulated core cellular processes like inflammation and mitochondrial metabolism?

The potentially most novel result comes from the authors’ analysis of NDDs by phenotype, which is a nice framework. However, I am not convinced by the link between seizure-associated NDDs and cerebellar Purkinje cells. The authors support their conclusion based on expression of these genes in the adult brain—not the developing brain. LHX1 and LHX5 are associated with GABAergic neuron development throughout the midbrain (perhaps even the whole brainstem). It therefore seems more plausible to me that these genes induce disease by affecting GABAergic neuron development more broadly.

Additionally, the authors claim that the “cerebellar inhibitory neurons” supercluster comprises mainly Purkinje neurons, but their supplementary figure states they are unable to find key markers for these genes. This supercluster might not contain Purkinje neurons at all unless the dataset enriched for them, because these neurons are rare. As far as I know, they should not dominate this supercluster.

Less important:
- Figure 1B: What does the x-axis represent? I did not find this plot intuitive.
- Figure 2A: I’m not sure it’s fair to call these genes “shared” across NDDs. This heatmap shows that the genes are not dysregulated in all samples, and behave very differently across samples. Perhaps “common” is more appropriate?
- Figure 2B: How can we see that these genes are not specific to one disease that happens to dominate the data? Perhaps disease information, e.g. a bar, could be added to the plot.
- Figure 2D: How were the odds calculated? Is this GSEA?
- Figure 3D: The text suggests that some of these pathways are “specific” to particular NDDs. What do the authors use to determine specificity (a statistical test, % samples significantly enriched, etc.)? I found this hard to assess myself. For example, the immune response is aberrant in four DMD and RTT samples, although the authors state this pathway is unique to DS.

Version 1:

Reviewer comments:

Reviewer #1

(Remarks to the Author)
Dear authors,

The authors followed all the recommendations and I can see that they put a lot of effort in this revision, which significantly improved the manuscript.
I am happy with the changes included and have no further suggestions.

Reviewer #2

(Remarks to the Author)
The authors have addressed all my concerns and the manuscript is much stronger - thank you and well done!

Reviewer #3

(Remarks to the Author)
My comments have mostly been addressed. I appreciate the authors' clarifications to the text and figures. I do have one follow-up comment regarding point 2, the use of adult vs developmental data: I did not mean the data used to discover the association. My apologies for being unclear. Rather I refer to the paragraph below, where I was surprised that developmental datasets were not mentioned given the nature of the study:

"The brain RNA expression data from the Human Protein Atlas revealed that LHX1 expression is highly specific to the cerebellum (Figure 4E). This is in contrast to LHX5 which is also abundantly expressed in the hypothalamus and medulla oblongata (Supplementary Figure S12). Moreover, single cell RNA (scRNA) sequencing data of the cerebellar vermis (Human Brain Atlas8 version 1.0) revealed that LHX1 is almost exclusively transcribed in cerebellar neurons, particularly in those of the cerebellar inhibitory supercluster (Figure 4F)."

Focusing on the exclusivity of LHX1 to adult cerebellum seems misleading to me. LHX1 and LHX5 are abundantly expressed during development, across all midbrain (and hindbrain?) GABAergic neurons. I think that is worth nothing. You should be able to check their expression in publicly available whole-brain developmental datasets, e.g. from the Linnarsson group. Of course, this does not change that Purkinje neurons warrant further study.

Open Access This Peer Review File is licensed under a Creative Commons Attribution 4.0 International License, which permits use, sharing, adaptation, distribution and reproduction in any medium or format, as long as you give appropriate credit to the original author(s) and the source, provide a link to the Creative Commons license, and indicate if changes were

made.
In cases where reviewers are anonymous, credit should be given to 'Anonymous Referee' and the source.
The images or other third party material in this Peer Review File are included in the article's Creative Commons license, unless indicated otherwise in a credit line to the material. If material is not included in the article's Creative Commons license and your intended use is not permitted by statutory regulation or exceeds the permitted use, you will need to obtain permission directly from the copyright holder.
To view a copy of this license, visit https://creativecommons.org/licenses/by/4.0/

Response to the reviewers

Integrative analysis of 115 transcriptomic studies decodes the molecular landscape of neurodevelopmental disorders

Reviewer #1 (Remarks to the Author):

1. As the authors decided to include a graphical abstract, I suggest that the authors improve the detail of the information provided, as for instance, the second part of the image is not informative, particullarly what the authors mean with "common alterations".

Thank you for your comment. We adjusted the graphical abstract by changing "common alterations" to "alterations across disorders" (see graphical abstract below).

[image redacted]

2. Can you supply a supplementary table demonstrating that the datasets cluster better by cell type than by disease, as this is a very interesting observation and a key result of your analysis, and I think that the data shown in figure 1D is not sufficient to illustrate this conclusion.

Thank you for your suggestion. We have now performed hierarchical cluster to further demonstrate that the datasets cluster by tissue/cell type and not by disease. We added this figure to the supplementary materials (see Supplementary Figure S2 below).

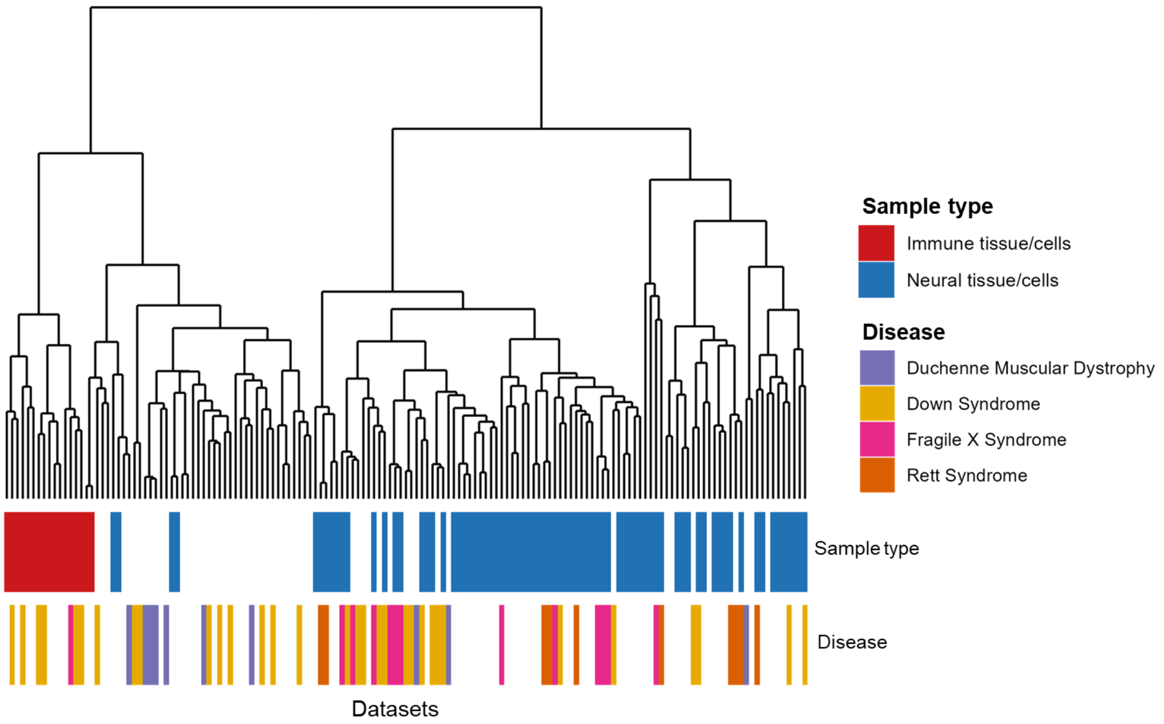

Supplementary Figure S2. Dendrogram showing the similarity between the 151 datasets. The distance between the datasets was calculated using the Spearman correlation of the gene's P values. The gene's P values were calculated for each dataset through the differential expression analysis of neurodevelopmental disorders cases versus controls. Ward D2 was used as linkage method.

3. The authors perform Gene Set Enrichment Analysis on the 151 datasets to find Gene Ontology Biological Process terms with a differential expression profile across the NDDs. The 30 GO-BP terms that reached statistical significance were shown and were gouped in 4 different subgroups; However, you don't explain how you perform this subgrouping. Additionally, I would like that you add a table showing which are the genes implicated in the 30 GO terms, also because it is important to have a picture of how many genes define your 4 groups of biological processes and the level of signifficance, to understand how genes are contributing to define BPs. It is not clear to me from the data shown that the gene APP that you refer as being largely alterated across several datasets is not related with sinaptyc signalling as well as SCNA, since APP function is associated to neuronal migration. I wonder if you have enough evidence to descriminate between the groups 3 and 4, as it is possible that the GO-BPs associated to mitochondrial function are related with neuronal development. I here suggest that you also compute the GO molecular functions and GO celular components to clarify this question, and the interpretation and discussion of the subgrouping presented by the authors.

Thank you for your comments. The subgrouping was defined based on the differential expression profile of the GO terms across the datasets by performing hierarchical clustering (Euclidean distance and Ward D2 linkage) on the signed -$\log_{10}$ P values. This is described in the Methods section (page 17):

“The top 30 GO-BP terms were clustered based on their signed -$\log_{10}$ P value using hierarchical clustering (Euclidean distance and Ward D2 linkage).”

These subgroups are not only distinct in terms of their differential expression profile, but also in terms of overlapping genes. To illustrate this, we have now added a GO similarity network to the supplementary materials (see Supplementary Figure S3B below), showing that the number of common genes between the subgroups is limited. This analysis provides additional evidence for discriminating between groups 3 and 4. Furthermore, we have added GO-MF and GO-CC analysis to the supplementary materials (see Supplementary Figure S4 below).

As suggested, we have now also provided a visual overview of the differential expression profile of the individual genes in the 30 GO terms (see Supplementary Figure S3A below) as well as a supplementary table that specifies which genes are included in each GO term with the $\log_2$FCs across all datasets (Supplementary Dataset S2).

Finally, as shown in Figure 2C, the *APP* and *SCNA* genes are both annotated to the synaptic signaling GO term (this annotation is based on the GO database). From the genes annotated to this GO term, *APP* was the most frequently differentially expressed gene. *SCNA* was less frequently differentially expressed than *APP*. However, *SCNA* was the most frequently differentially expressed gene annotated to the oxidative phosphorylation GO term. To clarify this in our manuscript, we rephrased the text in the Results (page 6) and the legend of Figure 2B (page 8):

Adjusted text in Results section (page 6):

“Among the genes in the inflammation-, RNA translation-, ATP synthesis-, and synaptic signaling-related GO-BP terms, HLA-B, SH3BGRL, APP, and SNCA, respectively, were differentially expressed in the largest number of datasets, exemplifying gene-level transcriptomic alterations across NDDs (…)”

Adjusted text in the legend of Figure 2B (page 8)

“B) To exemplify gene-level transcriptomic alterations across 151 NDD datasets, this panel shows the $\log_2$FC profile of the most frequently differentially expressed gene for the GO-BP terms related to inflammation, RNA translation, ATP synthesis, and synaptic signaling (i.e., HLA-B, SH3BGRL, APP, and SNCA, respectively).”

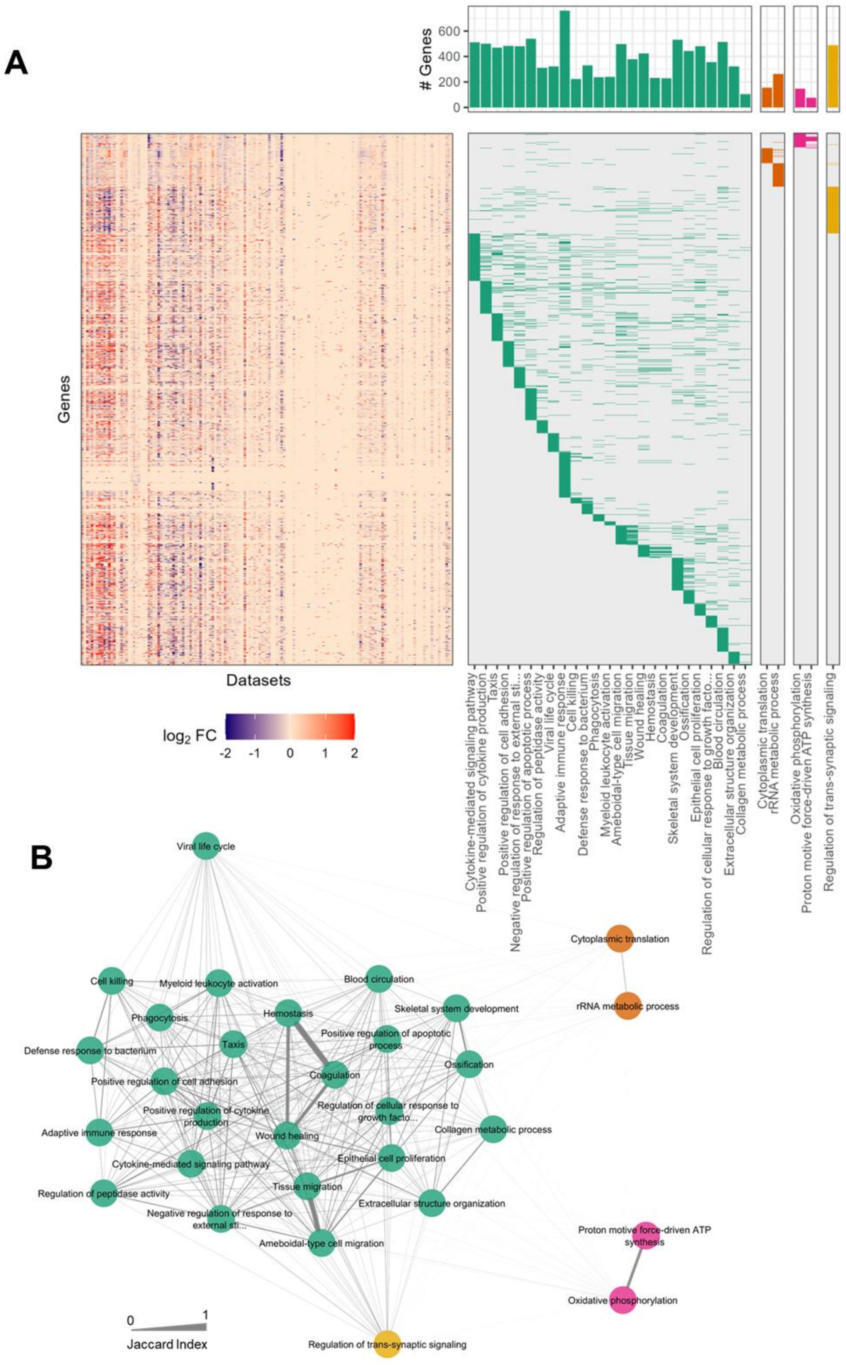

Supplementary Figure S3. Overview of the 30 GO-BP terms that were identified to exhibit transcriptomic changes across the NDD spectrum (see Figure 2 in manuscript). **A)** Heatmap of the log_2FCs for the genes in the 30 GO-BP terms across the 151 datasets (left panel). The right panel shows how many genes are associated to each GO-BP term (top) and to which GO-BP term(s) each gene is associated (bottom). An overview of which genes are included in each GO term is provided in Supplementary Dataset S2. **B)** Network diagram showing the similarity between the 30 GO-BP terms. The similarity (edge width) between two GO-BP terms is expressed as the Jaccard Index (*i.e.*, size of the intersection divided by the size of the union).

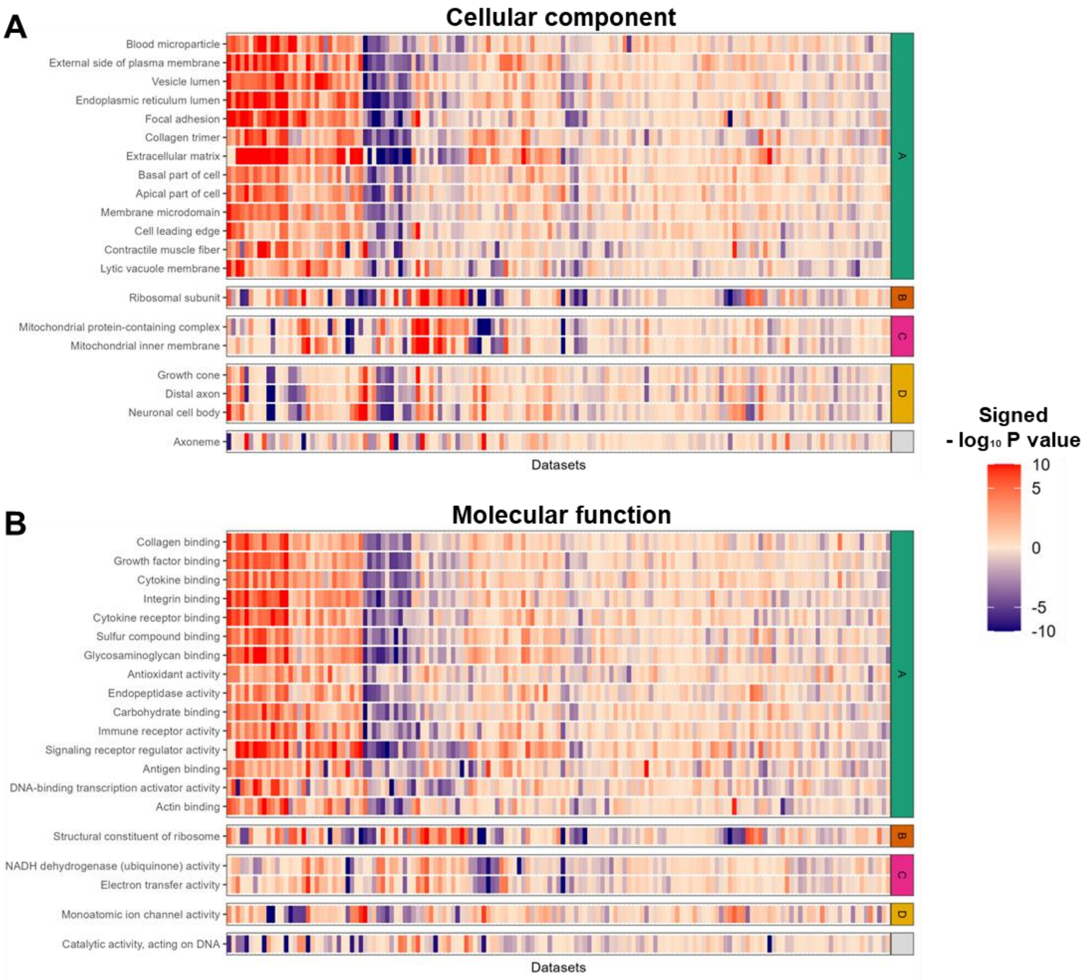

Supplementary Figure S4. Common alterations between the neurodevelopmental disorders (NDDs). **A)** Heatmap of the enrichment of the top 20 most frequently enriched Gene Ontology – Cellular component (GO-CC) terms across the 151 NDD datasets. **B)** Heatmap of the enrichment of the top 20 most frequently enriched Gene Ontology – Molecular function (GO-MF) terms across the 151 NDD datasets.

4. The identification of disease-specific transcriptomic changes for DMD, DS, FXS, and RTT is quite interesting since these disorders are usually mainly discussed in the context of single-gene mutations. In this context, add some lines in the discussion on how your analysis adds to previous evidence of gene association to such monogenic syndromic disorders.

Thank you for your comment. We have now modified and added the following text to the Discussion (page 14):

"In our disease-specific meta-analysis, we identified several transcriptomic markers for DMD, DS, FXS, and RTT (Figure 3). The identification of these transcriptionally dysregulated genes may provide insights into key pathways downstream of their underlying genetic alterations. Among the identified markers, the upregulation of PFN2 and ITGB4 across the DMD and RTT datasets, respectively, may relate to known disease mechanisms, which are elaborated upon in the paragraphs below."

5. As the authors found that differential expression of cerebellar Purkinje cell layer formation was strongly correlated with seizure-associated NDDs, I suggest adding some sentences on the importance of the GABAergic signalling involving cerebellar Purkinje cells and about the evidence linking this pathway with inhibitory/excitatory imbalance, seizures and disease (for a review in this subject: https://www.sciencedirect.com/science/article/pii/S0969996123002735). The focus of this final discussion can be shifted towards the implications of the abnormal cerebellar Purkinje cell layer formation to the the inhibitory signalling, and you can highlight that your analysis suggests an alternative mechanism for this dysregulation, other than the dysregulation of neurotransmission or receptor pathways, here involving cerebellar Purkinje cell layer formation (-as the authors also mention) and probably result from differential gene expression from the genes LHX1 and LHX5.

Thank you for your suggestions. We now more elaborately discuss the link between Purkinje cell formation and seizures in NDD in our Discussion (page 15):

"(…) Specifically, Purkinje cells are GABAergic neurons that provide inhibitory stimuli to the deep cerebellar nuclei[41]. Through this inhibitory pathway, cerebellar Purkinje cells prevent a seizure-causing hyperexcitability state of the cerebellum. In neurodegenerative disorders, seizures are thought to result from Purkinje cell degeneration or disruptions in Purkinje cell activity[41,42]. Our analysis suggests an alternative mechanism in neurodevelopmental disorders, where the dysregulated LHX1 and LHX5 expression may contribute to seizure onset by impairing the formation of the Purkinje cell pool."

Reviewer #2 (Remarks to the Author):

1. Firstly, the authors highlight significantly increased changes in imprinted gene expression than expected by chance. I really like the fact the authors have focused on these genes and the rationale for this is robust. However, I could not find the list of imprinted genes used in this study. There are many contrasting ideas as to the extent of genomic imprinting, with some using differential transcriptomic studies to suggest there are >2000. However, more conservative (and probably accurate) estimates indicate ~250 in humans.The fact that the authors have a comparable list of 195 suggests they have used conservative lists like others more recently (10.1186/s12864-022-08986-8). But how this list was generated is not apparent.

Thank you for your comment. We indeed used the more conservative list from geneimprint.com. We now specifically mention this source in the Methods (page 17-18):

*"For this, the Fisher's exact test was applied to assess whether previously reported or predicted imprinted genes (**obtained from geneimprint.com on December 5, 2023**) have higher odds of differential expression compared to non-imprinted genes."*

2. Another issue is highlighted by the fact that one of the key findings was that ageing-related transcriptomic changes were a significant group inthe analysis across NDDs. This is interesting but, as the authors do mention, is this driven by the large representation of Downs Syndrome (DS) samples, which represent >20% of all the samples analysed? We know that DS have accelerated ageing and often develop early onset Alzheimer's disease - could there over-representation be a factor for this finding more generally? This raises the question of whether there was any weighting given to normalise the contribution of the four highly represented disorders (RTT, DS, FXS and DMD)? This isn't clear from the analysis methods, but may of course be something to consider at least.

Thank you for your question and suggestion. We acknowledge that the imbalance between NDDs could introduce a bias in the analysis of the "common alterations". Therefore, we checked whether the transcriptomic changes in the identified GO-BP terms (*i.e.,* age-related processes) correlate with any of the four most common NDD (*i.e.,* RTT, DS, FXS, DMD) or eight neurological phenotypes (*i.e.,* intellectual disability, hypotonia, global developmental delay, microcephaly, gait ataxia, autism/autistic behavior, seizure, and scoliosis). However, we did not find any significant associations (Supplementary Figure S5), meaning that the age-related changes are not driven by a specific NDD or phenotype. For clarity, we rephrased this description in the Result section (page 6):

"Notably, enrichment of the GO-BP terms (i.e., -log10 P value of the GSEA) was not associated with any specific NDD or neurological phenotype (independent two-group Mann-Whitney U Test, FDR-adj. P value > 0.05, Supplementary Figure S5), suggesting that these transcriptomic changes are not driven by a specific disorder- or phenotype but instead are shared across the NDD spectrum."

3. Finally, although the details of the sources (i.e tissue) of the RNA-seq data are available in the supplementary material, I think these need some discussion in the main text. A key factor for me when mining these data is that most are not tissue samples at all, but are in fact in vitro derived lines - 91/151 (~60%) were iPSC / ESC / cell line - derived samples. I appreciate that this is not unexpected, but it could be clearer. Moreover, how much of an impact does this have on the findings? For instance, there is a literature indicating that imprinted gene expression is not 100% faithful in in vitro systems - how much is this driving the changes in imprinted genes seen here? Similarly, only 56 of the datasets are on CNS sample-types (Neuron / Brain / NPC) - how does this affect the patterns (and relevance) of GO-terms identified? The slightly disappointing thing is that this could have been explored here, as there is possibly the Power to analyse subsets of these data to test these questions. For instance, are the patterns of change broadly similar if all in vitro system samples are excluded?

Thank you for your questions. Imprinting might indeed not be 100% faithful in *in vitro* cell lines. However, since the cases and controls are from the same samples, there should not be a bias towards higher or lower expression of these genes. Nevertheless, to test this, we have now performed analysis of genomic imprinting separately for datasets from *in vitro* cell cultures *(e.g.,* iPSC/ESC-derived, fibroblast, and immortalized cell lines) (n = 119) and non-*in vitro* models (*e.g.,* blood, biopsy, and post-mortem tissue) (n = 32) and added this analysis to the manuscript. From this analysis (see Supplementary Figure S9 below), it can be seen that, in 75% of the non-*in vitro* datasets, imprinted genes are more likely differentially expressed than non-imprinted genes (*i.e.,* $\log_2$ OR > 0), which is more than expected by chance (permutation P value = 0.003). The preferential dysregulation of imprinted genes occurs in 83% of the *in vitro* datasets (permutation P value < 0.001). Although the effect size is slightly larger in *in vitro* datasets, there is no association between model system (*i.e., in vitro* versus non-*in vitro*) and the number of datasets with preferential differential expression of imprinted genes (Chi-square test P value = 0.42).

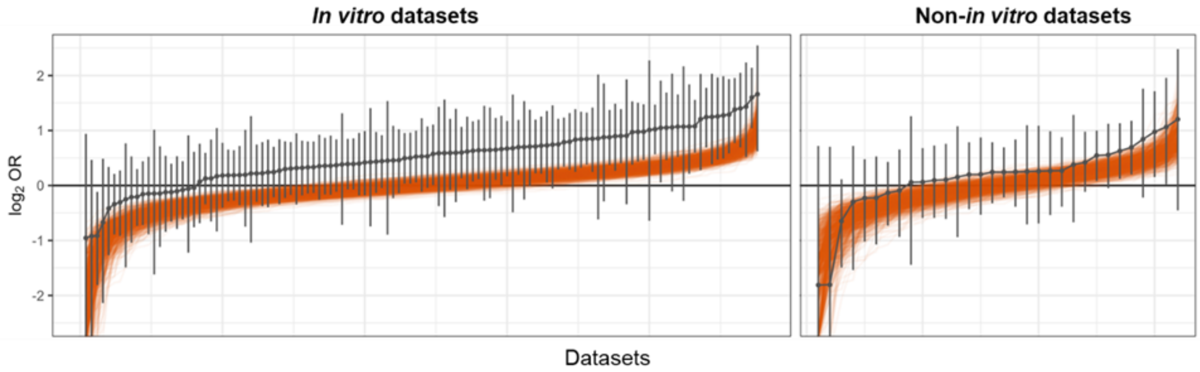

Supplementary Figure S9. The $\log_2$ Odds Ratio ($\log_2$ OR) and the 95% confidence interval (C.I) of differential expression (P value < 0.05) of imprinted versus non-imprinted genes. Imprinted genes have higher odds of being differentially expressed than non-imprinted genes ($\log_2$ OR > 0) in 99 of the 119 *in vitro* datasets (*e.g.,* stem cell-derived, fibroblast, and immortalized cell lines) and 24 of the 32 non-*in vitro* datasets (*e.g.,* blood, biopsy, and post-mortem tissues) datasets. The orange lines show the ORs of 1000 random gene sets with the same size and expression profile as the imprinted genes. The higher odds of differential expression of imprinted genes occurs more than expected by chance both in *in vitro* (permutation P value < 0.001) and non-*in vitro* (permutation P value = 0.003) datasets.

Regarding the GO terms, we did not find any association between the sample type (immune, neural, or other tissues/cell types) and the enrichment of the GO terms (see Supplementary Figure S5 below). This shows that the enrichment of the identified GO terms is not specific to a sample type. Moreover, compared to our initial manuscript, we have now also tested whether the differential expression of the GO terms is associated with *in vitro* and non-*in vitro* model systems and added this to our supplementary materials (see Supplementary Figure S5 below). This analysis shows that the enrichment of the GO terms is not specific to *a* model system.

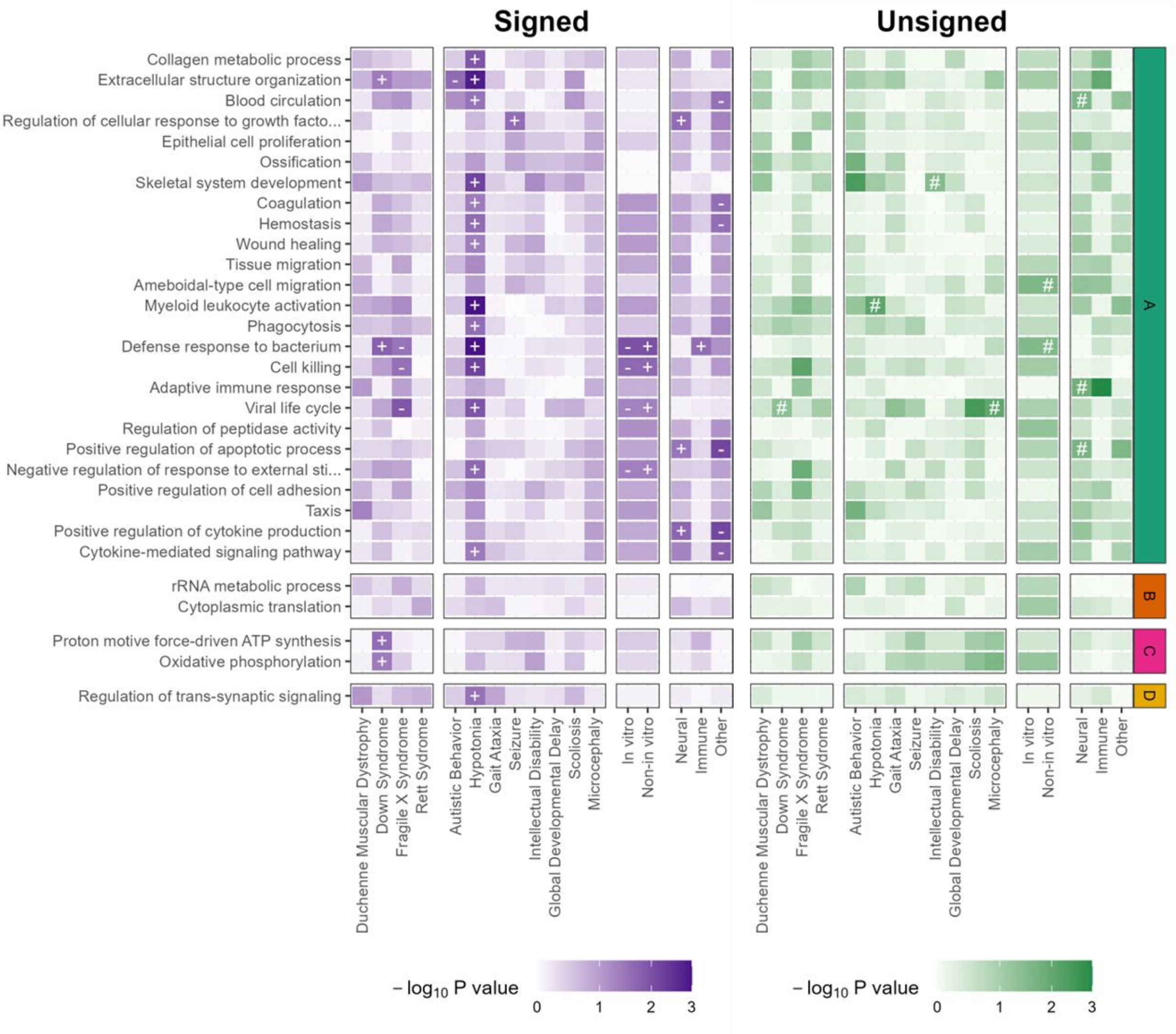

Supplementary Figure S5. Association of the top 30 GO-BP terms with specific neurodevelopmental disorders, neurological phenotypes, model systems, and sample types. Independent two-group Mann-Whitney U Test was used to find the GO-BP terms (*i.e.*, signed and unsigned -$\log_{10}$ P value) that are significantly associated with any of the four neurodevelopmental disorders *(i.e.*, Duchenne muscular dystrophy, Down Syndrome, Fragile X Syndrome, Rett Syndrome), eight neurological phenotypes (*i.e.*, intellectual disability, hypotonia, global developmental delay, microcephaly, gait ataxia, autism/autistic behavior, seizure, and scoliosis), (non)-*in vitro* model systems, and sample types (*i.e.,* immune, neural, and other cell types/tissues). After FDR-adjustment, none of the GO-BP terms correlate with any of the phenotypes, disorders, model systems, or sample types. In the left panel, nominally significantly higher levels of positive enrichment (positive -$\log_{10}$ P value) in the associated datasets are indicated by "+", while nominally significantly higher levels of negative enrichment (negative -$\log_{10}$ P value) in the associated datasets are indicated by "-". In the right panel, nominally significantly higher levels of enrichment (unsigned -$\log_{10}$ P value) in the associated datasets are indicated by "#". P value < 0.05 is considered nominally significant.

Reviewer #3 (Remarks to the Author):

1. I enjoyed reading the paper. Beautiful figures, clear and straightforward writing style, and the analysis seems sound. I also appreciate that the authors created a database for exploring their analysis. However, the findings do not seem very novel. The authors state themselves that imprinted genes, synaptic signaling, mitochondrial processes, and inflammatory responses are already associated with NDDs. I also do not find wholly convincing that their results point to a connection with neurodegenerative disease. Will not diseases with very different pathologies anyway converge on dysregulated core cellular processes like inflammation and mitochondrial metabolism?

Thank you for your comments and questions. Our study includes several novel aspects. For instance, the connection between imprinted genes and NDDs has always been based on specific examples (*e.g.,* Angelman and Prader-Willi Syndrome syndrome) but has never been systematically evaluated. Our systematic evaluation of the transcriptomic profile of imprinted genes is novel and addresses this gap. We have now described this novelty more clearly in the Discussion (Page 12):

"(…) Until now, the link between NDDs and imprinted genes has been based on these specific examples. Our findings, however, provide the first systematic evidence for the dysregulation of imprinted genes across a wide range of neurodevelopmental disorders. This widespread dysregulation can be explained by the essential roles of imprinted genes in the regulation of neural proliferation, differentiation, and migration[7]. Particularly, any alteration in these tightly regulated processes may impair normal brain development, giving rise to NDD phenotypes."

The associations of *PFN2*, *ZNF22*, *SRBD1*, and *ITGB4* with DMD, DS, FXS, and RTT, respectively (as identified in the disorder-specific meta-analysis) have, to our knowledge, neither been described before. The same is true for the link between *LHX1* dysregulation and seizures in NDDs (as identified in the phenotype-specific analysis). Together, we believe that our findings include multiple novel aspects that are of value for the scientific community.

Regarding the connection to neurodegenerative diseases, we acknowledge that various pathologies converge on core cellular processes such as inflammation and mitochondrial dysfunction. This convergence might not be surprising as many diseases, including those with distinct etiologies, have been linked to a state of accelerated ageing. In addition to the dysregulation of these ageing-related processes, the potential link between neurodevelopmental and neurodegenerative disorders was further highlighted by the dysregulation of key neurodegenerative disease-related genes, *APP* and *SCNA,* across the NDDs. In light of these findings and the hypothesis-generating nature of our study, we think it is worth discussing the potential overlap in the molecular pathophysiology between neurodevelopmental and neurodegenerative disorders as it may direct further studies. Nevertheless, to address the need for further research on the topic, we have now added the following text to the Discussion (page 13):

"(…) Together, our findings highlight the need for dedicated analyses assessing the possible overlap in the molecular pathophysiology of neurodevelopmental and neurodegenerative disorders, and its potential for therapeutic interventions."

2. The potentially most novel result comes from the authors' analysis of NDDs by phenotype, which is a nice framework. However, I am not convinced by the link between seizure-associated NDDs and cerebellar Purkinje cells. The authors support their conclusion based on expression of these genes in the adult brain—not the developing brain. LHX1 and LHX5 are associated with GABAergic neuron development throughout the midbrain (perhaps even the whole brainstem). It therefore seems more plausible to me that these genes induce disease by affecting GABAergic neuron development more broadly.

Thank you for your comments. It is important to note that our findings are not only based on the expression in the adult brain, as many of the included datasets are from iPSC-derived NPCs and neurons which might actually be more representative of the developing brain (see Mertens *et al.*, https://doi.org/10.1038/nrn.2016.46). Notably, the datasets with the largest expression changes in cerebellar Purkinje cell layer formation and *LHX1* (*i.e.,* the labeled datasets in Figure 4B and D) are all from stem cell-derived model systems.

Although *LHX1* and *LHX5* show the strongest dysregulation, other genes involved in Purkinje cell layer formation are also more dysregulated in seizure-associated NDDs than in NDDs without seizures (see positive $\log_2$ OR of these genes in Figure 4C). This is not the case for other *LHX1/5*-associated gene ontologies, which exhibit a transcriptional dysregulation that is less specific to seizure-associated NDDs (see figure below). This is why we in particular hypothesize that there is a link between seizure-associated NDDs and cerebellar Purkinje cells.

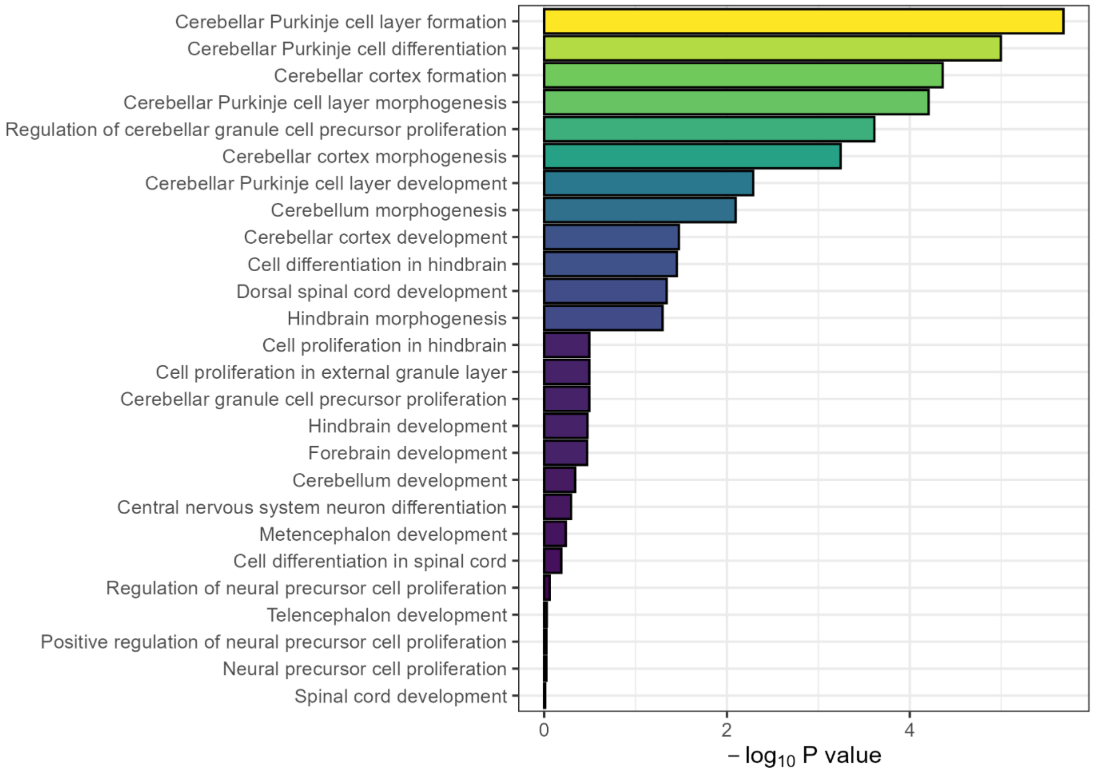

Figure A1. Association of the enrichment of LHX1/5-associated GO terms with seizure phenotype. For the GO terms that include both *LHX1* and *LHX5*, the two-group Mann-Whitney U Test was applied to test for association between the GO enrichment ($-\log_{10}$ P value) and seizure phenotype. The significance of this association is shown in this figure. It can be seen that Cerebellar Purkinje cell layer formation and related processes are more strongly associated with a seizure phenotype than other neurodevelopmental GO terms (*e.g.,* hindbrain development).

Nevertheless, as our study is a hypothesis-generating study, we do not want to make any hard claims on the involvement of Purkinje cell layer formation in seizure-associated NDDs. Instead, we want to bring this process up as a potential avenue for future research and we, therefore, added the following text to the Discussion (page 15):

“However, it should be noted that LHX1 and LHX5 have multifaceted roles during the development of the brain, including regulation of axonal guidance and neural survival[43,44]. Some of these may also contribute to seizures in NDDs—an aspect that warrants further investigation in dedicated future studies.”

3. Additionally, the authors claim that the “cerebellar inhibitory neurons” supercluster comprises mainly Purkinje neurons, but their supplementary figure states they are unable to find key markers for these genes. This supercluster might not contain Purkinje neurons at all unless the dataset enriched for them, because these neurons are rare. As far as I know, they should not dominate this supercluster.

Thank you for your comment. In our Results (page 10), we have now removed the text claiming the domination of the cerebellar inhibitory supercluster by Purkinje cells.

Less important:

- Figure 1B: What does the x-axis represent? I did not find this plot intuitive.

Figure 1B shows how many datasets (y-axis) there are with a specific number of cases and controls (x-axis). To enhance the clarity of this figure, we have now phrased this more elaborately in the legend of Figure 1B:

“The y-axis shows the number of datasets with the specified number of cases and controls on the x-axis. Most datasets include only 3 or 4 cases and controls, while only a few datasets include more than 10.”

Furthermore, we have changed the y-axis label to “# Datasets” instead of “# Statistical comparisons” for consistency across figures.

- Figure 2A: I’m not sure it’s fair to call these genes “shared” across NDDs. This heatmap shows that the genes are not dysregulated in all samples, and behave very differently across samples. Perhaps “common” is more appropriate?

Thank you for your suggestion. In our revised manuscript, we now refer to these changes as “common” changes.

- Figure 2B: How can we see that these genes are not specific to one disease that happens to dominate the data? Perhaps disease information, e.g. a bar, could be added to the plot.

Thank you for your suggestion. When we add a bar with disease information to the plot, it is still difficult to accurately see the associations of these genes with a specific disease. Instead,

similar to our analysis of the GO terms (Supplementary Figure 5), we have now performed an additional statistical analysis to check whether the log_2FC profile of these genes correlate with any specific NDD, neurological phenotype, sample type, or model system. We have added the results of this analysis to Supplementary Figure S6 and the legend of Figure 2B.

Legend Figure 2B:

"(…) The log_2FC profiles of HLA-B, SH3BGRL, and SCNA are not associated with any specific disorder, phenotype, sample type, or model system (independent two-group Mann-Whitney U Test, FDR-adj. P value > 0.05, Supplementary Figure S6). However, because of its location on chromosome 21, APP exhibits higher log_2FCs in Down syndrome than in any other disorder (independent two-group Mann-Whitney U Test, FDR-adj. P value = 3.9e-7). Nevertheless, after excluding Down syndrome datasets, APP is still among the top 10% most frequently differentially expressed genes in the "Regulation of trans-synaptic signaling" GO term."

Supplementary Figure S6:

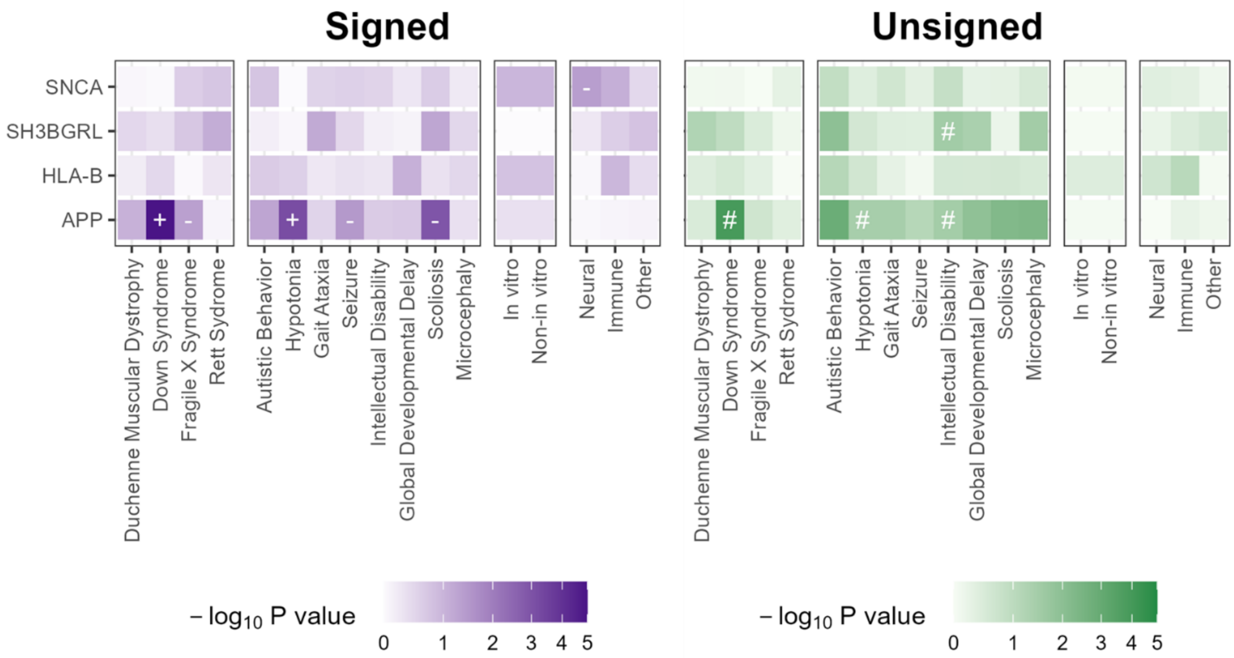

Supplementary Figure S6. Association of *SNCA*, *SH3BGRL*, *HLA-B*, and *APP* with neurodevelopmental disorders, neurological phenotypes, model systems, and sample types. Independent two-group Mann-Whitney U Test was used to identify whether the log_2FC (signed) and absolute log_2FC (unsigned) profile of these four genes are significantly associated with any of the four neurodevelopmental disorders *(i.e.*, Duchenne muscular dystrophy, Down Syndrome, Fragile X Syndrome, Rett Syndrome), eight neurological phenotypes (*i.e.*, intellectual disability, hypotonia, global developmental delay, microcephaly, gait ataxia, autism/autistic behavior, seizure, and scoliosis), (non)-*in vitro* model systems, and sample types (*i.e.,* immune, neural, and other cell types/tissues). Because of its location on chromosome 21, *APP* exhibits higher log_2FCs in Down syndrome than in any other disease (independent two-group Mann-Whitney U Test, FDR-adj. P value = 3.9e-7). After FDR-adjustment, the log_2FC profiles of *HLA-B*, *SH3BGRL*, and *SCNA* are not associated with any specific disorder, phenotype, sample type, or model system (independent two-group Mann-Whitney U Test, FDR-adj. P value > 0.05). In the left panel, nominally significantly higher log_2FCs in the associated datasets are indicated by "+", while nominally significantly lower log_2FCs in the associated datasets are indicated by "-". In the right panel, nominally significantly higher absolute log_2FCs in the associated datasets are indicated by "#". P value < 0.05 is considered nominally significant.

- Figure 2D: How were the odds calculated? Is this GSEA?

The odds and their confidence interval were calculated with the Fisher's exact test. We described this in the Methods (page 17-18), but we have now also added this to the legend of Figure 2D (page 8):

"D) The log_2 Odds Ratio (log_2 OR) and the 95% confidence interval (C.I) of differential expression (P value < 0.05) of imprinted versus non-imprinted genes ***as calculated by the Fisher's exact test****."*

- Figure 3D: The text suggests that some of these pathways are "specific" to particular NDDs. What do the authors use to determine specificity (a statistical test, % samples significantly enriched, etc.)? I found this hard to assess myself. For example, the immune response is aberrant in four DMD and RTT samples, although the authors state this pathway is unique to DS.

Thank you for your comment. In this context, we used "specific" to indicate that we performed the meta-analysis per disease (*i.e.,* disease-specific meta-analysis). However, we acknowledge that using the term "specific" can be confusing as we did not assess whether the identified changes are unique to a specific disorder. Therefore, in our revised manuscript, we now refer to this as "disorder-**associated** alterations" and removed the term "unique" from the description of our Results as follows (page 8):

"Additionally, by performing GSEA, several processes with differential expression profiles in DMD, DS, FXS, and RTT were found (Figure 3D). For instance, axonogenesis was downregulated in RTT and humoral immune response was upregulated in DS."

Response to the reviewers

Integrative analysis of 115 transcriptomic studies decodes the molecular landscape of neurodevelopmental disorders

Reviewer #1
The authors followed all the recommendations and I can see that they put a lot of effort in this revision, which significantly improved the manuscript.
I am happy with the changes included and have no further suggestions.

Thank you. No further revision necessary.

Reviewer #2
The authors have addressed all my concerns and the manuscript is much stronger - thank you and well done!

Thank you. No further revision necessary.

Reviewer #3
My comments have mostly been addressed. I appreciate the authors' clarifications to the text and figures. I do have one follow-up comment regarding point 2, the use of adult vs developmental data: I did not mean the data used to discover the association. My apologies for being unclear. Rather I refer to the paragraph below, where I was surprised that developmental datasets were not mentioned given the nature of the study:

"The brain RNA expression data from the Human Protein Atlas revealed that LHX1 expression is highly specific to the cerebellum (Figure 4E). This is in contrast to LHX5 which is also abundantly expressed in the hypothalamus and medulla oblongata (Supplementary Figure S12). Moreover, single cell RNA (scRNA) sequencing data of the cerebellar vermis (Human Brain Atlas8 version 1.0) revealed that LHX1 is almost exclusively transcribed in cerebellar neurons, particularly in those of the cerebellar inhibitory supercluster (Figure 4F)."

Focusing on the exclusivity of LHX1 to adult cerebellum seems misleading to me. LHX1 and LHX5 are abundantly expressed during development, across all midbrain (and hindbrain?) GABAergic neurons. I think that is worth nothing. You should be able to check their expression in publicly available whole-brain developmental datasets, e.g. from the Linnarsson group. Of course, this does not change that Purkinje neurons warrant further study.

Thank you for your comment. In line with your suggestion, we have now used scRNA-seq data from first trimester developing human brain (Linnarsson group, https://doi-org.mu.idm.oclc.org/10.1126/science.adf1226) to look at *LHX1* and *LHX5* expression during development. Indeed, *LHX1* and *LHX5* are expressed across the midbrain and hindbrain. We have, therefore, added the following text and Supplementary Figure S12 to the manuscript:

Results section (page 6):

“Publicly available single cell RNA sequencing (scRNA-seq) data from first trimester developing human brain[10] showed that during early neurodevelopment, both LHX1 and LHX5 are mostly expressed in the hindbrain and midbrain regions (Supplementary Figure S12). In the adult brain (Human Protein Atlas[11] v24.0), LHX1 expression is highly specific to the cerebellum (Figure 4E). This is in contrast to LHX5 which is also abundantly expressed in the hypothalamus and medulla oblongata (Supplementary Figure S13). Moreover, scRNA-seq data of the cerebellar vermis (Human Brain Atlas[8] v1.0) revealed that LHX1 is almost exclusively transcribed in cerebellar neurons, particularly in those of the cerebellar inhibitory supercluster (Figure 4F).”

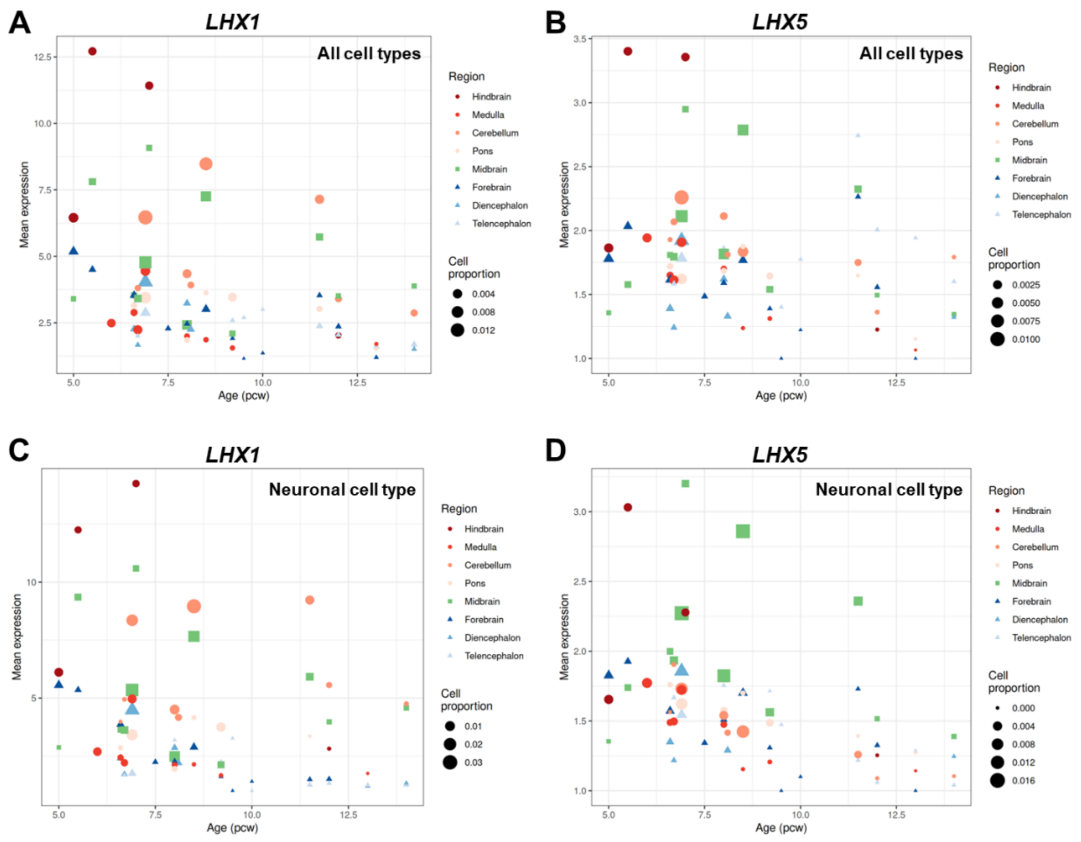

Supplementary Figure S12. Expression of *LHX1* and *LHX5* in first trimester developing human brain. The processed single cell RNA expression and annotations from Braun *et al.*[1] were downloaded from CZ CELLxGENE Discover (https://cellxgene.cziscience.com/collections/4d8fed08-2d6d-4692-b5ea-464f1d072077). The age in postconceptional weeks (pcw) is plotted against the mean expression of *LHX1* and *LHX5* in the expressing cell fraction. The expressing cell fraction, as indicated by the point size, is the proportion of cells with a read count of at least one. **A)** and **B)** show the expression of *LHX1* and *LHX5* in all cell types combined, while **C)** and **D)** show the expression of *LHX1* and *LHX5* in neurons.

Methods section (page 14):

“(…) the single cell expression profile of LHX1 and LHX5 during the first trimester of the developing human brain was assessed using the processed scRNA-seq data from Braun et al.[10]. “
